# Tailoring the morphology of AIEgen fluorescent nanoparticles for optimal cellular uptake and imaging efficacy†
†Electronic supplementary information (ESI) available: Experimental methods and other additional data. See DOI: 10.1039/c7sc05130a


**DOI:** 10.1039/c7sc05130a

**Published:** 2018-01-17

**Authors:** Jianxu Zhang, Bin Xu, Wenjing Tian, Zhigang Xie

**Affiliations:** a State Key Laboratory of Polymer Physics and Chemistry , Changchun Institute of Applied Chemistry , Chinese Academy of Sciences , 5625 Renmin Street , Changchun , Jilin 130022 , P. R. China . Email: xiez@ciac.ac.cn; b State Key Laboratory of Supramolecular Structure and Materials , Jilin University , Changchun , 130012 Jilin , P. R. China . Email: xubin@jlu.edu.cn; c University of Chinese Academy of Sciences , Beijing 100049 , P. R. China

## Abstract

The rational design of robust fluorescent organic materials for long-term cell tracing is still challenging, and aggregation-caused quenching of emission is a big limitation of this strategy. Organic dyes with aggregation-induced emission (AIE) can effectively address this problem. Herein, AIEgen-containing nanoparticles, with different morphologies and emission, were prepared by assembling amphiphilic copolymers with an AIEgen. We compared the physical and chemical properties of rod-like and spherical nanoparticles, particularly investigating the effects of the shape on internalization and the imaging effect. The formulated nanoparticles exhibit advantageous features, such as a large Stokes shift, robust stability in physiological conditions, strong fluorescent emission, and photobleaching resistance. Interestingly, the rod-like nanoparticles were internalized more efficiently than their spherical counterparts, and their strong green fluorescence can still be clearly observed even after 15 days *in vitro* and *in vivo*. This work demonstrates the great potential of regulating the morphology of nanoparticles to obtain an ideal biological function.

## Abstract 2

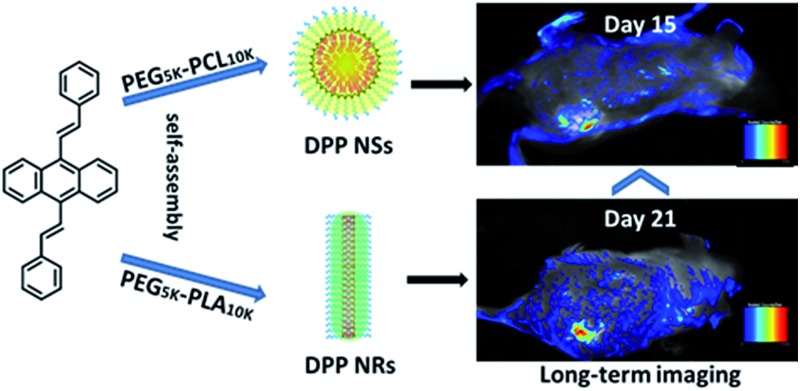
We have demonstrated a new approach for regulating the morphology and emission of AIE-active organic nanoparticles by assembling different amphiphilic copolymers.

## Introduction

Bioimaging has become a powerful tool in biological research because it makes the process of diagnosis easy, and offers a unique approach to visualizing the morphological details of cells and tissues.^1^–^4^ In recent years, researchers have increasingly paid attention to various imaging techniques, such as computed tomography,^5^–^7^ magnetic resonance imaging,^6^,^8^,^9^ fluorescence imaging,^10^–^14^ positron emission tomography,^15^ and photoacoustic tomography.^7^,^8^,^16^ Among these imaging techniques, fluorescence imaging possesses indispensable and versatile features including minimal invasiveness, high contrast, good spatial and temporal resolution, high sensitivity and quantitative information at subcellular levels.^17^–^19^ Over the past few decades various fluorescent materials have been developed, such as fluorescent proteins,^20^–^22^ quantum dots,^23^,^24^ carbon dots,^25^–^27^ organic dyes,^28^–^30^ and fluorescent organic nanoparticles (FONPs).^18^,^31^–^37^ Among these, FONPs remain relatively unexplored for biomedical application, which is mainly due to two limitations: (1) their inherent fluorescence quenching,^38^,^39^ which is common for most organic fluorophores during their aggregation in aqueous media; and (2) their uncontrolled morphology and cellular behavior.^40^–^42^ Fortunately, since the concept of aggregation-induced emission (AIE) was originally reported by Tang *et al.*,^43^ a large number of new AIE materials^32^,^33^,^44^–^50^ have been explored for their various applications.^33^,^44^,^45^,^47^,^51^–^53^ AIE molecules possess very bright fluorescence in the aggregated state,^55^,^56^ which makes them perfect for bioimaging.^36^,^54^ Recently, many kinds of AIE-based nanoparticle have been developed through physical cladding or covalent binding.^34^,^48^ During the process of fabricating AIE nanoparticles, polymers could protect the payload and tune the physicochemical properties, and therefore play an important role.^34^ However, determining the nature of the AIE molecules or polymers to finely tune the morphologies of the AIE nanoparticles is uncharted territory and a great challenge.

Understanding the interactions of nanoparticles with living cells is a key point to engineering ideal nanoparticles for bioimaging.^41^,^57^,^58^ Recent literature suggests that alterations in the parameters of nanoparticles, such as the surface functionalization, size, geometry, and charge, can significantly affect the pathway of endocytosis and the intracellular fate of the nanoparticles.^40^,^59^–^62^ Among these parameters, the geometrical shape of the nanoparticles has not received enough attention to date, partly because of the great challenge of designing nonspherical nanoparticles.^60^,^63^,^64^ Recently, there have been several observations about the design of optimal nanoparticles for imaging and therapy. For example, Zhou *et al.* demonstrated that rod-like micelles exhibited accelerated cellular internalization compared to sphere-shaped micelles.^65^ He *et al.* revealed that an appropriate increase of the aspect ratio would facilitate the cellular uptake of mesoporous silica nanoparticles.^66^ Our previous work has also showed similar results.^67^ In general, rod-shaped particles appear to be more favorably engulfed compared to their spherical counterparts.^68^

**Scheme 1:**
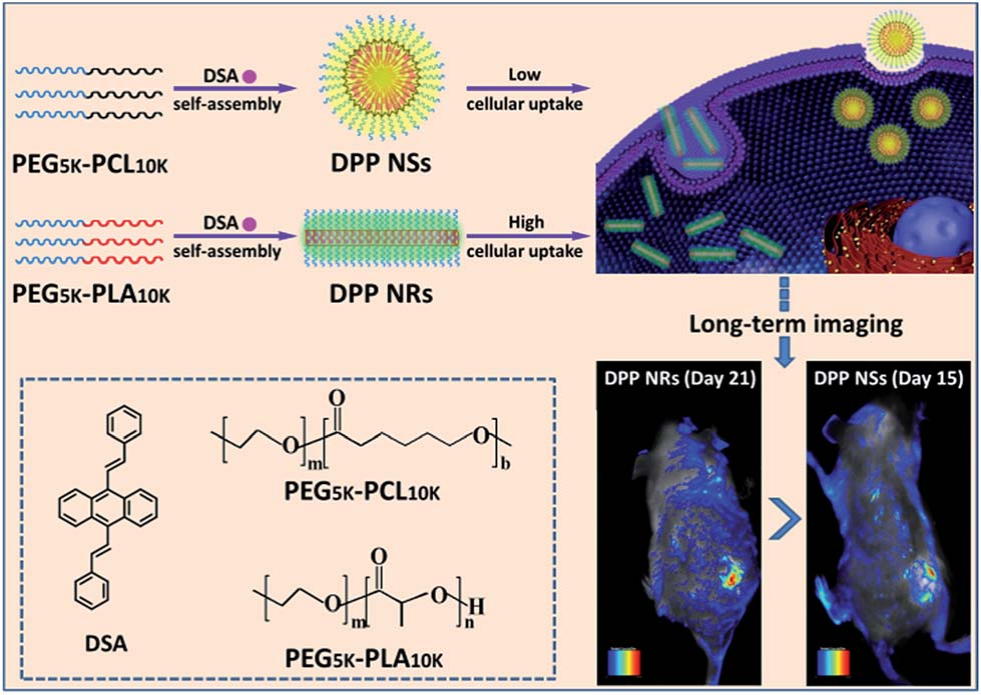
An illustration showing the preparation of spherical and rod-like AIEgen nanoparticles from DSA, and the comparison of their cellular uptake and imaging *in vitro* and *in vivo*.

In this work, we have developed a universal approach to produce a group of AIEgen fluorescent nanoparticles with different shapes, and investigated their cellular uptake (Scheme 1). The differently shaped particles were readily internalized in HeLa cells, and the rod-like micelles had faster internalization rates than their spherical counterparts, leading to a better imaging effect *in vitro* and *in vivo*.

## Results and discussion

**Fig. 1:**
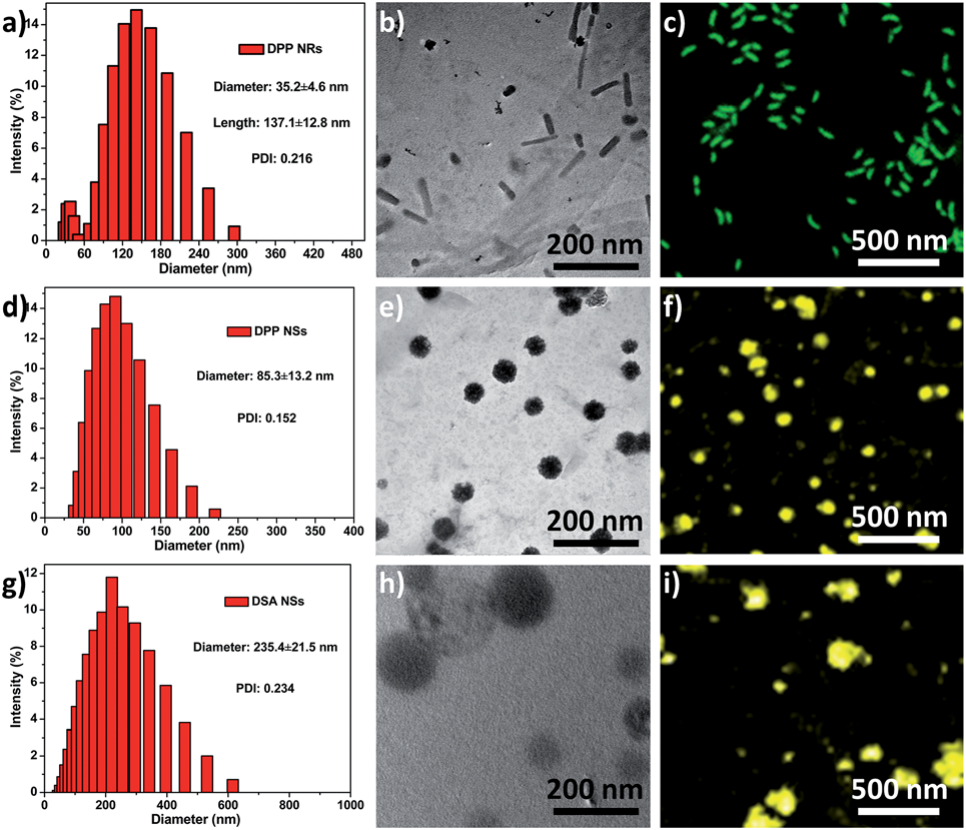
Size distribution, TEM images and CLSM images of the (a–c) DPP NRs; (d–f) DPP NSs; (g–i) DSA NSs. Scale bars in all TEM images are 200 nm. Scale bars in all CLSM images are 500 nm.

Herein, the rod-like micelles were obtained when we used poly(ethylene glycol)-*block*-poly(l-lactic acid) (PEG_5k_-PLA_10k_) to encapsulate 9,10-distyrylanthracene (DSA), but the spherical micelles were made using poly(ethylene glycol)-*block*-poly(caprolactone) (PEG_5k_-PCL_10k_). The rod-like and spherical nanoparticles were named as DPP NRs and DPP NSs, respectively. For comparison, we also prepared DSA nanoparticles (spherical, DSA NSs) through a nanoprecipitation method. We systematically compared the properties of the three AIE nanoparticles in detail. DSA was synthesized according to our previously reported procedures.^50^ The chemical structures of PEG_5k_-PLA_10k_ and PEG_5k_-PCL_10k_ were confirmed using ^1^H NMR (Fig. S1†). DPP NRs were prepared by adding a mixed THF solution of DSA and PEG_5k_-PLA_10k_ dropwise into water with vigorous stirring for 1 h, followed by dialysis to remove the residual THF. The DPP NSs were prepared by following the same protocol, but using PEG_5k_-PCL_10k_ instead of PEG_5k_-PLA_10k_. The DSA NSs were made in aqueous solution in the absence of polymer. The size distribution and morphologies of the DPP NRs/DPP NSs were characterized by dynamic light scattering (DLS), transmission electron microscopy (TEM) and confocal laser scanning microscopy (CLSM). As shown in Fig. 1, the DPP NRs were about 35.2 nm in diameter and about 137.1 nm in length, with a PDI value of 0.216. The DPP NSs and DSA NSs possessed an average size of 85.3 nm and 235.4 nm, and a PDI of 0.152 and 0.234, respectively. TEM images revealed the smooth rod-like morphology of the DPP NRs, while the DPP NSs and DSA NSs were spherical, but the spherical shape of the DSA NSs was not homogeneous. The DLS and TEM results of the DPP NRs and DPP NSs were different from those of the micelles of PEG_5k_-PLA_10k_ and PEG_5k_-PCL_10k_, respectively (Fig. S2†). In addition, the critical micelle concentration of PEG_5k_-PCL_10k_ was lower than that of PEG_5k_-PLA_10k_ (Fig. S3†). Before observing the morphologies of the nanoparticles by CLSM, the AIE effect of DSA, the DPP NRs and the DPP NSs was confirmed, as shown in Fig. S4 and S5.† As shown in Fig. 1c, the DPP NRs were in the form of well-defined and monodisperse nanorods with green fluorescence, while the DPP NSs (Fig. 1f) and DSA NSs (Fig. 1i) exhibited spherical morphologies with yellow fluorescence. These results indicate that AIEgen nanoparticles (AIE NPs) with different morphologies emit different fluorescence.

**Fig. 2:**
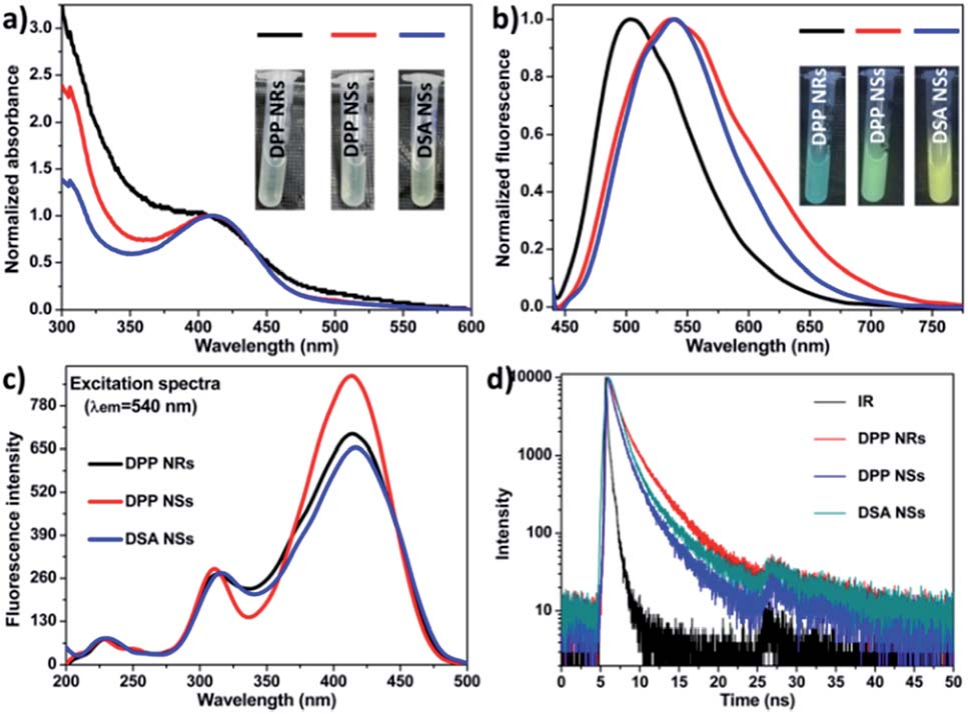
(a) Absorbance spectra of the DPP NRs, DPP NSs and DSA NSs. Insets: photographs of the DPP NRs, DPP NSs and DSA NSs under room light. (b) PL (excited at 410 nm) spectra of the DPP NRs, DPP NSs and DSA NSs. Insets: photographs of the DPP NRs, DPP NSs and DSA NSs under UV light. (c) Excitation spectra of the DPP NRs, DPP NSs and DSA NSs. (d) Time-resolved decay profiles of the DPP NRs, DPP NSs and DSA NSs.

We compared their optical properties by UV-Vis absorption and photoluminescence spectra. The concentration of DSA in all samples was the same, which was adjusted according to the UV-vis standard curves (Fig. S6†). As shown in Fig. 2, the absorption peak of the DPP NRs was slightly blue-shifted compared with that of the DPP NSs, and their photographs under room light were almost the same (inset Fig. 2a). For their PL spectra (Fig. 2b), the maximum emission of the DPP NRs appears at 500 nm, while those of the DPP NSs and DSA NSs were at 540 nm, which is consistent with the noticeable color changes shown in Fig. 1 and the inset in Fig. 2b. Moreover, the three AIE nanoparticles possess large Stokes shifts of about 100 nm, which greatly minimizes self-absorption and thus improves the signal-to-noise ratio for imaging. Although the DPP NRs showed different fluorescent emission to that exhibited by the DPP NSs and DSA NSs, their excitation spectra were almost the same (Fig. 2c). The quantum yield of the DPP NRs, DPP NSs and DSA NSs in water was 58.67%, 67.71% and 60.39%, respectively, which are much higher than that of DSA in THF (28.41%) (Table S1†). The fluorescence lifetimes of the DPP NRs, DPP NSs and DSA NSs were 2.02, 1.26 and 1.66 ns, respectively, which are shorter than that of free DSA (2.77 ns) (Fig. 2d and S7†). All these data are collected in Table S1.† These results indicate that the AIE NPs are totally different formulations with AIE molecules.

**Fig. 3:**
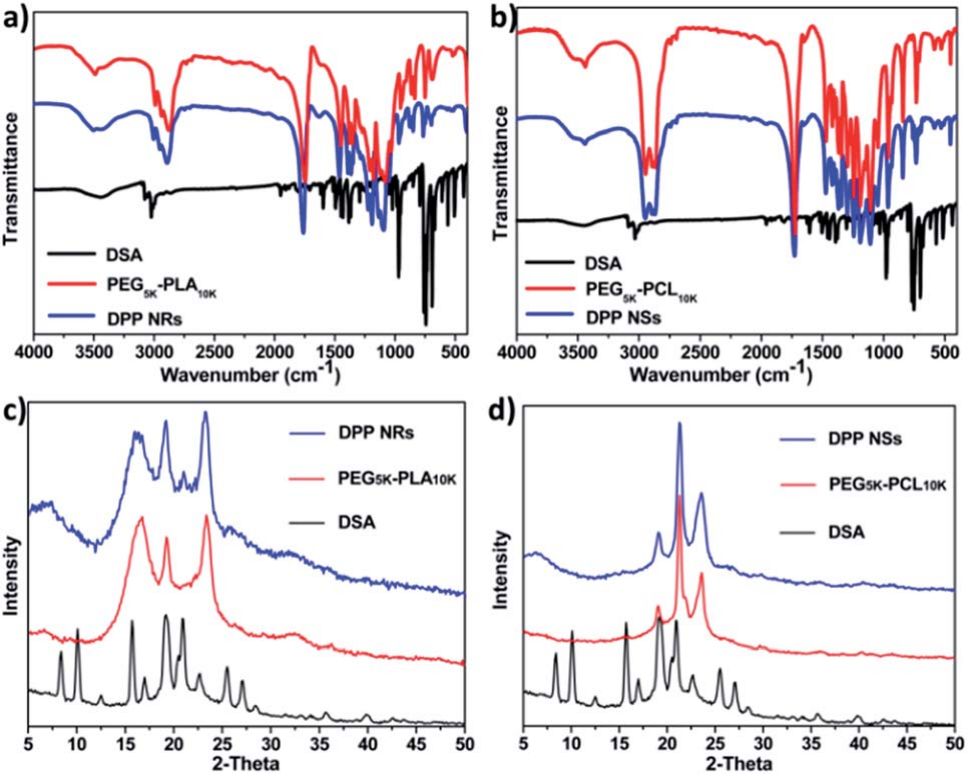
(a) FTIR spectra of as-prepared DSA (black line), PEG_5k_-PLA_10k_ (red line) and DPP NRs (blue line) in the solid state. (b) FTIR spectra of as-prepared DSA (black line), PEG_5k_-PCL_10k_ (red line) and DPP NSs (blue line) in the solid state. (c) The powder X-ray diffractogram of the DPP NRs (blue line), PEG_5k_-PLA_10k_ (red line) and DSA (black line). (d) The powder X-ray diffractogram of the DPP NSs (blue line), PEG_5k_-PCL_10k_ (red line) and DSA (black line).

In order to reveal the assembly mechanism, we used Fourier transform infrared (FT-IR) spectroscopy and powder X-ray diffraction (PXRD) to further study the aggregates formed between the copolymers and DSA. As shown in Fig. 3a, the spectrum of the DPP NRs was red shifted compared with that of PEG_5k_-PLA_10k_, indicating that the interactions between PEG_5k_-PLA_10k_ and DSA were strong, presumably as a result of the synergy of noncovalent supramolecular interactions including π–π stacking and hydrophobic interactions. In contrast, the spectrum of the DPP NSs was almost the same as that of PEG_5k_-PCL_10k_, revealing that the interactions between PEG_5k_-PLA_10k_ and DSA were weak (Fig. 3b). Furthermore, the PXRD of the freeze-dried DPP NRs showed well-resolved peaks, which is different to the situation with PEG_5k_-PLA_10k_. Meanwhile, the PXRD of the DPP NSs is similar to that of PEG_5k_-PCL_10k_ (Fig. 3c and d). These results demonstrated that the DSA molecules in the DPP NRs possess higher crystallinity than those of the DPP NSs, which leads to the different optical properties exhibited between the DPP NRs and DPP NSs. Moreover, to study whether this strategy can be a general approach to regulate the morphology of AIEgen-encapsulated organic nanoparticles, we used these two copolymers to form assemblies with three other AIEgens. As shown in Fig. S8,† only AIE3@PEG-PLA showed a rod shape, indicating that the structure of the AIEgens also played an important role in this study. In addition, we also studied the effect that the copolymer concentration has on the morphology of the micelles. As shown in Fig. S9,† the shape of the DPP NRs changed obviously with an increase in the concentration of copolymer, while still keeping a general rod-like shape. As a control, the DPP NSs changed slightly. These results suggested that this assembling strategy is special for DSA and its derivatives.

Excellent stability is essential for retaining the shape and function of the nanoparticles in blood circulation. Here, we evaluated the stabilities of the AIE NPs by monitoring the size distribution, absorbance and fluorescence spectra in various conditions. As displayed in Fig. S10,† the DPP NRs and DPP NSs stored in Dulbecco’s modified Eagle’s medium (DMEM) with 10% fetal calf serum (FBS) and 1% penicillin/streptomycin exhibited unchanged sizes and size distributions after 5 days. In contrast, the size of the DSA NSs changed obviously, and their PDI value increased in 2 days (Fig. S11†). Moreover, the appearance of all the nanoparticles was still transparent after five days, without obvious aggregates or precipitates (Fig. S12†). Furthermore, we studied the effect of human serum albumin (HSA) on the morphology of the micelles during storage. As shown in Fig. S13,† the size distribution of the DPP NRs and DPP NSs only increased slightly, which was mainly due to the absorption of the protein on the surface of the micelles. The above results indicated that the DPP NRs and DPP NSs could keep a stable nanostructure under physiological conditions.

**Fig. 4:**
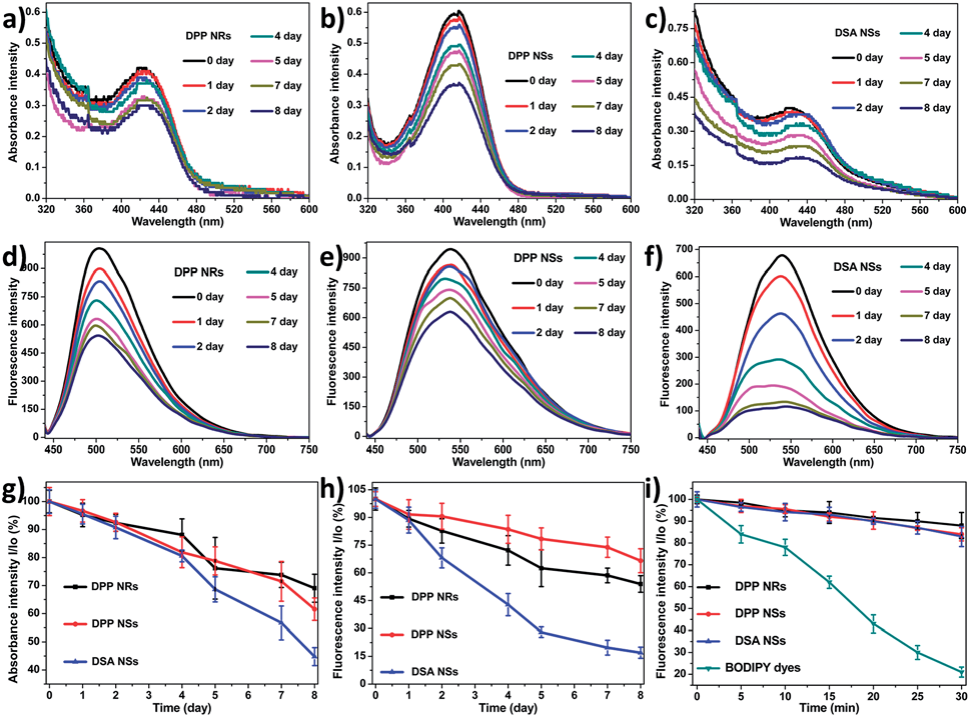
The absorbance intensity of the (a) DPP NRs, (b) DPP NSs and (c) DSA NSs over eight days. The fluorescence intensity of the (d) DPP NRs, (e) DPP NSs and (f) DSA NSs over eight days. (g) The stability of the absorbance intensity and (h) fluorescence intensity of the DPP NRs, DPP NSs and DSA NSs over eight days. Data represent mean values ± standard deviation, *n* = 3. (i) Photostability of the DPP NRs, DPP NSs, DSA NSs and BODIPY dyes under continuous scanning at 488 nm. *I*_0_ is the initial PL intensity, while *I* is that of the corresponding sample after a designated time interval.

Furthermore, we collected the absorbance and fluorescence spectra of the AIE NPs in aqueous solution over 7 days. As depicted in Fig. 4a–h, the absorbances of the DPP NRs and DPP NSs all decreased slowly and retained more than 60% of the original value within one week, while that of the DSA NSs decreased significantly and was reduced to 42.5% of the original value. Changes of the fluorescence intensity gave similar results. These spectral results illustrate that both the DPP NRs and DPP NSs possess good physical and optical stability, which is favorable for biomedical applications. The photostability of the three AIE NPs was investigated by monitoring the fluorescence intensity upon continuous laser irradiation. As shown in Fig. 4i, after continuous laser irradiation at 488 nm for 30 min, the fluorescence intensity of the three AIE NPs decreased only slightly and maintained about 90% of their initial value. Furthermore, we monitored the green fluorescence signals from human cervical carcinoma (HeLa) cells pretreated with the three AIE NPs under laser irradiation for 30 min (Fig. S14†). There was no obvious bleaching of the fluorescence after 30 min of laser irradiation. For direct comparison, we also studied the photostability of BODIPY dyes (BDP), which are believed to possess robust photostabilities, under the same conditions.^67^ As shown in Fig. 4i, the fluorescence intensity of the BDP maintained only about 20% of the initial value after continuous laser irradiation. Moreover, the fluorescence intensity of BDP in HeLa cells rapidly diminished and became negligible due to severe photobleaching (Fig. S14†). The above results suggest that the DPP NRs and DPP NSs possess excellent physical and optical stability.

Biocompatibility is imperative for the use of fluorescent nanoparticles as bioimaging agents. We firstly studied the biocompatibility of DSA, PEG_5k_-PLA_10k_ and PEG_5k_-PCL_10k_ toward HeLa cells using an MTT (3-[4,5-dimethylthiazol-2-yl]-2,5-diphenyltetrazolium bromide) assay. As shown in Fig. S15a–c,† DSA, PEG_5k_-PLA_10k_ and PEG_5k_-PCL_10k_ all have low cytotoxicity toward HeLa cells at different concentrations after incubation for 24 h. Similarly, low cytotoxicity of the DPP NRs, DPP NSs and DSA NSs against cells was observed, and more than 90% of those cells were alive at different incubation concentrations. To further demonstrate visually the biocompatibility of the DPP NRs, DPP NSs and DSA NSs, we stained the cells with calcein-AM and propidium iodide to identify live (green) and dead/late apoptotic (red) cells, respectively. As exhibited in Fig. S15d–f,† no red fluorescence was observed for all of the samples, suggesting that the three AIE NPs have low cytotoxicity toward HeLa cells, which agrees well with the MTT experiments. Fig. S16† shows the morphology of the HeLa cells after incubation with different concentrations of the DPP NRs, DPP NSs and DSA NSs for 24 h; the cells maintain their normal morphology. These results confirmed the good biocompatibility of the DPP NRs, DPP NSs and DSA NSs.

Cellular uptake is necessary for nanomaterials to exert their functions, especially for live cell imaging. HeLa cells were used to investigate the cellular uptake of AIE NPs by CLSM. After incubating with DPP NRs at various concentrations for 2 h at 37 °C, cellular nuclei were dyed using 4,6-diamidino-2-phenylindole (DAPI). As presented in Fig. S17,† the homogeneous green fluorescence was located in the cytoplasm, suggesting that the DPP NRs can pass across the cell membrane into the cytoplasm. Moreover, the DPP NRs exhibit internalization by living cells in a concentration-dependent manner. The DPP NSs and DSA NSs showed similar results (Fig. S18 and S19†). Furthermore, the sub-cellular location of the internalized nanoparticles was carried out using lyso-tracker red. As shown in Fig. S20,† the AIE nanoparticles were mainly located within the endosome, and the co-localization of the DSA nanoparticles (green) with the endosome (red) produced an orange fluorescence in the merged images. All of these results confirmed that the DSA nanoparticles could be internalized effectively by cancer cells.

**Fig. 5:**
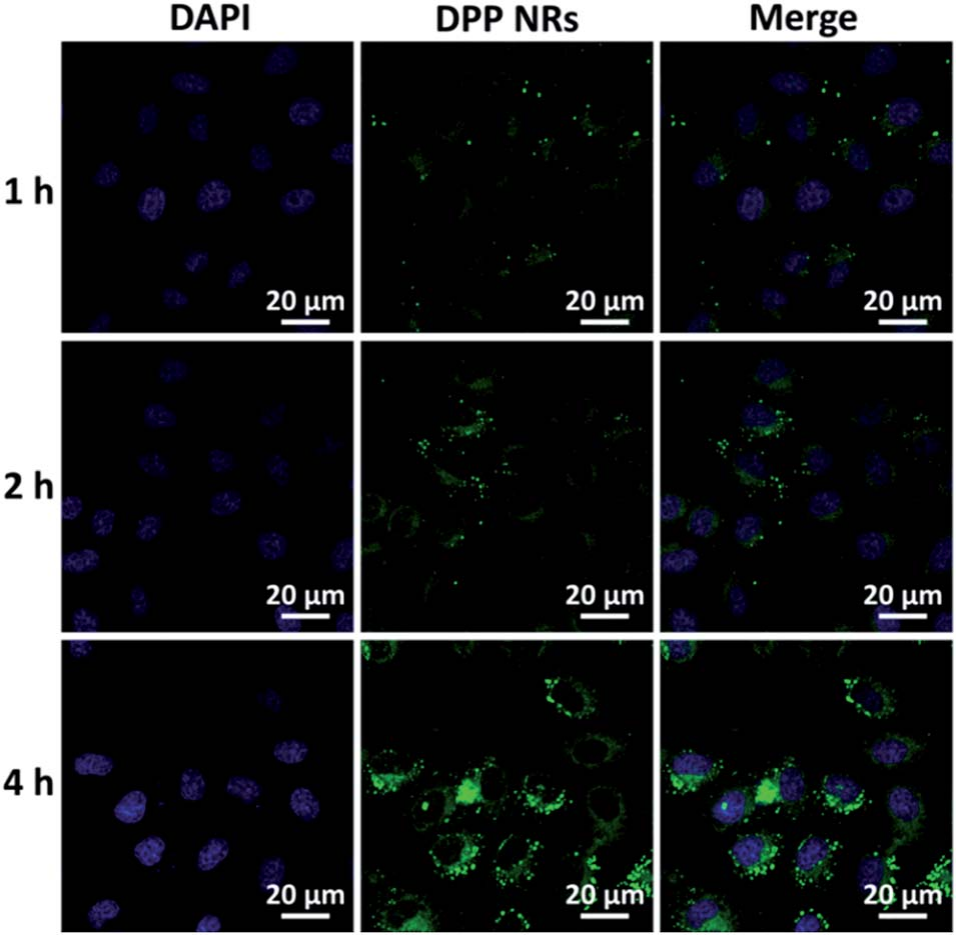
CLSM images of HeLa cells incubated with the DPP NRs for 1 h, 2 h and 4 h at 37 °C. Cells are viewed in the blue channel for DAPI, and the green channel for DSA. Scale bars represent 20 μm in all images.

**Fig. 6:**
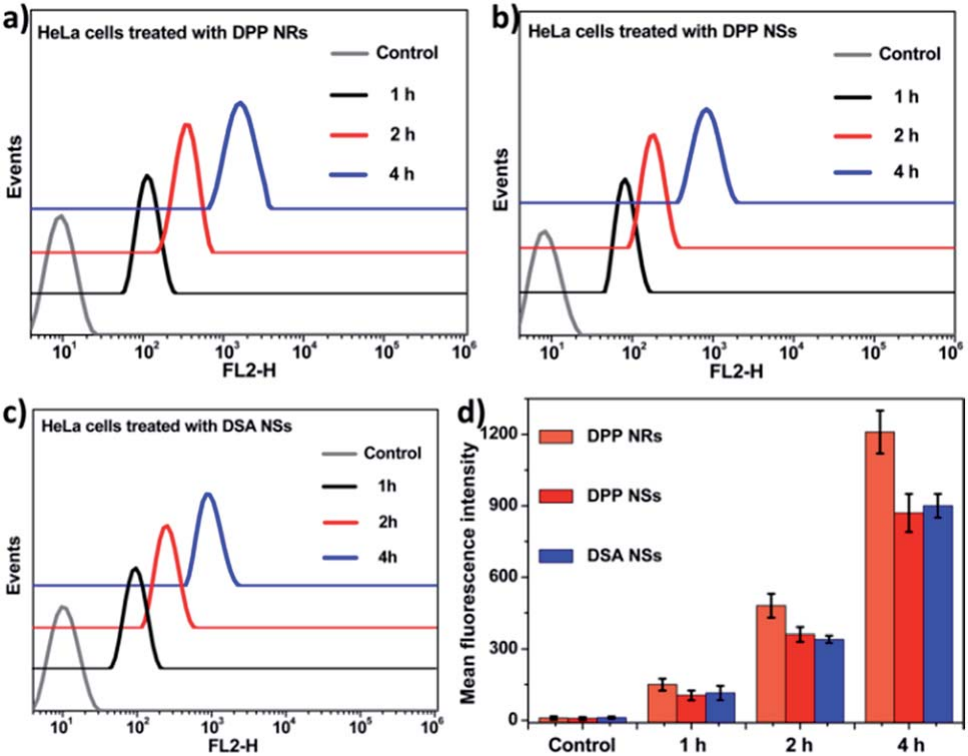
(a–c) Flow cytometry histograms of HeLa cells treated with the DPP NRs, DPP NSs and DSA NSs, and without treatment (control) for different time periods. (d) Quantitative analysis of (a–c); the data are presented as the mean values ± standard deviation, *n* = 3.

To evaluate the effects of the nanoparticle morphology on the cellular uptake efficiency, the HeLa cells were cultured with the DPP NRs, DPP NSs, and DSA NSs. As shown in Fig. 5, S21 and S22,† the intracellular fluorescent intensity increased gradually with the incubation time from 1 to 4 h, demonstrating that these AIE NPs had a sustained cellular uptake in HeLa cells. In addition, the DPP NRs exhibited the strongest green fluorescence, followed by the DSA NSs and DPP NSs, indicating that the DPP NRs were more easily internalized by cells compared with the DPP NSs and DSA NSs. Meanwhile, flow cytometry was employed to quantify the cellular uptake of the three AIE NPs. As shown in Fig. 6, the DPP NRs had relatively higher uptake efficiencies in comparison to the DPP NSs and DSA NSs. These results are in agreement with the CLSM results. In addition, we detected the cellular uptake efficiency of the NPs using UV-vis spectra (Fig. S23†). The absorbance of DSA extracted from the HeLa cells after different times increased from 1 to 4 h. The possible reason for the higher cellular uptake of the DPP NRs is that the rod-like nanoparticles have multivalent contact points with the cell membranes, resulting in stronger adhesions and an enhanced uptake relative to the spheres. To study the influence of the stability of the nanoparticles on the cellular uptake, we studied the cellular uptake of the DSA NSs in two conditions: freshly made, and after storing for 48 hours. As shown in Fig. S24,† the freshly made DSA NSs could pass into cells, but after 48 hours of storage time the DSA NSs were less able to enter cancer cells. Thus, we used the DPP NRs and DPP NSs for further studies in long-term imaging.

**Fig. 7:**
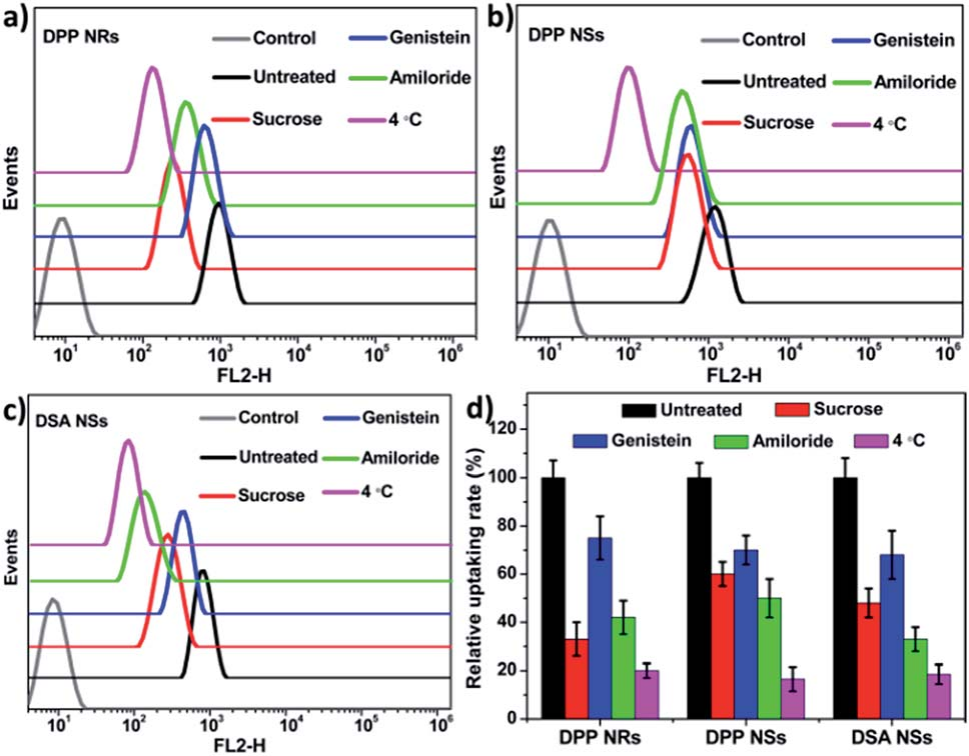
(a–c) Flow cytometry histograms of the HeLa cells treated with the DPP NRs, DPP NSs and DSA NSs, and without treatment (control), after treatment with different endocytic inhibitors. (d) Quantitative analysis of (a–c); the data are presented as the mean values ± standard deviation, *n* = 3.

To determine whether the AIE NPs with different morphologies are internalized *via* different endocytic pathways, we explored the uptake of polymer micelles by HeLa cells. Three types of inhibitor, namely sucrose, genistein and amiloride, were chosen to inhibit clathrin-mediated endocytosis, caveolae-mediated endocytosis, and macropinocytosis, respectively. Low temperature (4 °C) treatment was used to determine whether the endocytosis process was energy-dependent. To decrease the influence of the inhibitors on the cancer cells, experimental conditions were optimized according to previous publications.^65^ The flow cytometry results are shown in Fig. 7. The cells treated under low temperature all showed drastic decreases in the uptake of the AIE NPs, confirming an energy-dependent endocytosis process. The internalization at 37 °C showed that the AIE NPs with different morphologies show diverse uptake profiles. The uptake of the DPP NRs by the HeLa cells primarily occurred *via* the clathrin-mediated endocytic and micropinocytic pathways. It is more probable that macropinocytosis and/or phagocytosis are the mechanisms of uptake of the DSA NSs, due to their relatively large size. These observations suggest that the mechanisms of endocytosis are dependent on the shapes of the nanoparticles. A spherical nanoparticle has only one face that can interact with the cell surface, while rod-like nanoparticles have multiple faces with large variations in size in each dimension. Therefore, we concluded that the cellular uptake of nanoparticles with various shapes appears to be mediated by multiple pathways.

**Fig. 8:**
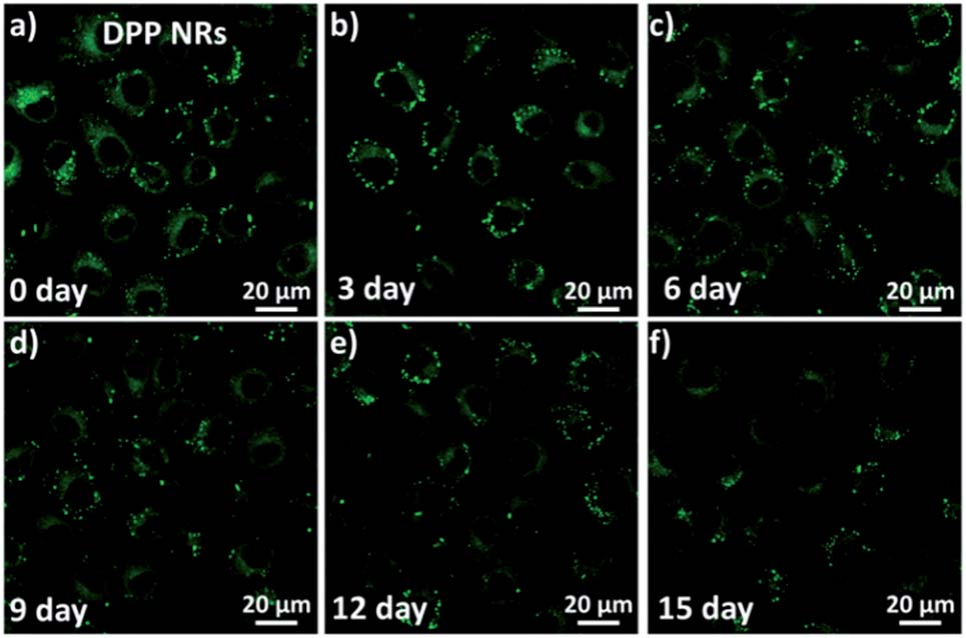
Long-term cell tracing images of the DPP NRs incubated at 37 °C for 6 h and then subcultured for designated time intervals including (a) day 0; (b) day 3; (c) day 6; (d) day 9; (e) day 12; and (f) day 15. Scale bars represent 20 μm in all images.

To investigate and compare the long-term cellular tracking capability of the DPP NRs and DPP NSs, we captured the fluorescence images after different incubation periods (Fig. 8). The HeLa cells were first incubated with the DPP NRs for 6 h at 37 °C (labelled as day 0). The treated cells were then subcultured for designated time intervals. For each cell passage, the old culture medium was extracted and the HeLa cells were washed with PBS twice to remove the DPP NRs present in the culture medium. At the initial stage (day 0), strong and bright green fluorescence from the DPP NRs can be clearly observed in Fig. 8. With the increase of incubation time (from day 3 to day 15), the green fluorescence gradually decreases because of cell proliferation. Interestingly, after 15 days of subculture, the green fluorescence from the DPP NRs was still clearly observed in the HeLa cells, which indicates that the DPP NRs can act as a fluorescent probe for long-term cellular imaging. By contrast, the DPP NSs (Fig. S25†) showed very weak fluorescence after 15 days of incubation. The DPP NRs showed stronger intracellular fluorescence than the DPP NSs at every time point (Fig. S26†). More importantly, this long-term imaging strategy is based on cellular proliferation and only needed a one-time addition of organic nanoprobes, rather than a continuous exogenous addition of imaging agents. All of these results indicate the superior cell tracing ability of the DPP NRs.

**Fig. 9:**
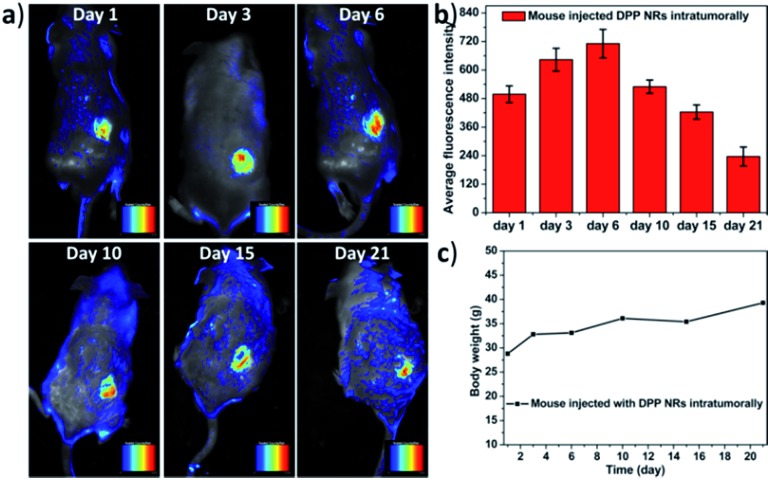
(a) Representative time-dependent *in vivo* fluorescence images of the tumor-bearing mouse that was intratumorally injected with the DPP NRs, from day 1 to day 21. (b) Time-dependent fluorescence intensity changes for the tumors. Data represent mean values ± standard deviation, *n* = 3. (c) Body weight of the mouse during the imaging time.

In order to further study the imaging capacity, the DPP NRs were intratumorally injected into tumor-bearing BALB/c mice, and then an *in vivo* optical imaging system was used to monitor the fluorescence over 21 days. As displayed in Fig. 9a and S27,† fluorescence from the site of the DPP NR injection could be readily detected after 21 days, while that of the DPP NSs showed very weak fluorescence after 15 days (Fig. S28 and S29†). The DPP NRs exhibited mild fluorescence at the injection site one day after injection. Over time, the fluorescence intensity gradually increased and showed the strongest fluorescence at day 6 (Fig. 9b). Then, the fluorescence intensity gradually decreased, but still could be readily detected after 21 days, suggesting that the DPP NRs still remained in the tumor, showing better imaging performance than the DPP NSs. Moreover, to further confirm that the fluorescence is from the DSA nanoparticles, we extracted the DSA from the tumor by tissue extraction with THF. As shown in Fig. S30,† the absorbance of DSA could be detected in the tumor extraction solution, and the absorbance intensity of the DPP NR group was stronger than that of the DPP NSs. The body weight of the mice in the two imaging groups gradually increased over time (Fig. 9c and S28c†), suggesting that the DPP NRs and DPP NSs had no distinct systemic toxicity. In addition, we also study the imaging capacity of the DSA nanoparticles by intravenous injection. As shown in Fig. S31,† fluorescence at the tumor site of the DPP NR group could be readily detected after 24 h. Over time, the fluorescence intensity gradually decreased, but could still be readily detected after 168 h, suggesting that the DPP NRs could accumulate in a tumor and show better imaging performance than the DPP NSs. Next, we studied the biodistribution of the DPP NRs and DPP NSs by detecting the fluorescence in the tumor and the major organs excised from the mice. As shown in Fig. S32 and S33,† at 48 h post-injection a strong fluorescence intensity could be clearly observed in the tumor, while weak fluorescence was observed in the organs, and the fluorescence of the DPP NR group was higher than that of the DPP NSs. These results indicated that the DPP NRs could accumulate and be retained around the tumor. The biosafety of the nanoparticles was evaluated by hematology analysis. As shown in Fig. S34,† the DPP NRs and DPP NSs all exhibited a negligible influence on the AST, ALT, and blood urea nitrogen (BUN) or creatinine (CREA) indexes, compared to those of the saline group. All of this information consistently demonstrated the good biocompatibility of the DSA nanoparticles. The above results all confirmed that the DPP NRs are potentially promising for *in vivo* imaging.

## Conclusions

In summary, stable AIEgen nanoparticles with different shapes were prepared, by the assembling of copolymers and AIE molecules, and used for noninvasive long-term imaging. The formulated nanoparticles exhibit superior physical and photo stability under physiological conditions. *In vitro* experiments have verified that these tailor-made AIE-active organic nanoparticles are biocompatible and are internalized through various pathways of cellular uptake. The long-term imaging ability was validated by *in vitro* and *in vivo* experiments. More importantly, the rod-like nanoparticles were significantly more internalized than the spherical particles, resulting in a better imaging effect. Our findings may provide useful information for the development of new strategies for the design of efficient AIEgen nanoparticles for bioimaging.

## Animal experiments

All animal experiments were performed in compliance with the NIH guidelines for the care and use of laboratory animals. The healthy male BALB/c mice (15–20 g) were purchased from the Laboratory Animal Center of Jilin University (Changchun, China) and maintained under required conditions. Use of them for this study was approved by the Animal Ethics Committee of Jilin University. Animal care and handling procedures were carried out according to the guidelines of the Regional Ethics Committee for Animal Experiments.

## Conflicts of interest

There are no conflicts to declare.

## Supplementary Material

Supplementary information
