# Supplementary material for: Tailoring the morphology of AIEgen fluorescent nanoparticles for optimal cellular uptake and imaging efficacy
Source: Chem Sci. 2018 Jan 17;9(9):2620–7. doi: 10.1039/c7sc05130a (PMC5892346; doi:10.1039/c7sc05130a)
Supplement: Supplementary file 1 [file SC-009-C7SC05130A-s001.pdf]

Electronic Supplementary Material (ESI) for Chemical Science.
This journal is © The Royal Society of Chemistry 2018

Electronic supplementary information

Tailoring Morphology of AIEgen Fluorescent Nanoparticles for Optimal Cellular Uptake and Imaging Efficacy

Jianxu Zhang,[a,c] Bin Xu,[b*] Wenjing Tian [b] and Zhigang Xie [a*]

1. Experimental Procedures

Materials. AIE luminogens used in this article were synthesized in Tian's group. MTT (3-[4,5-dimethylthiazol-2-yl]-2,5-diphenyltetrazoliumbromide) were purchased from Shanghai Beyotime Biotechnology Co., Ltd.. Cell viability (live dead cell staining) assay kit was purchased from Jiangsu KeyGEN Biotechnology Co., Ltd.. All of the other Chemicals and reagents were acquired from commercial sources without further purification, unless otherwise noted. All the solvents were purified according to the standard methods whenever needed. Milli-Q water was collected from a Milli-Q system (Millipore, USA).

Preparation of nanoparticles. DSA NSs were prepared using a reprecipitation method. In a typical procedure, the DSA solution (400 μL) was quickly dropwise dispersed into 4 mL of milli-Q water with vigorous stirring at room temperature for 30 min. Then the solution was dialyzed against Milli-Q water for 24 h, the cutoff molecular weight of the dialysis bags is 3500.

DPP NRs and DPP NSs were prepared using copolymer to assemble DSA in water. In a typical procedure, firstly, the copolymer solution (200 μL) was mixed with the DSA solution (400 μL), then the mixing solution was added dropwise to the 4 mL of milli-Q water with vigorous stirring at room temperature for 30 min. Then the solution was dialyzed against Milli-Q water for 24 h, the cutoff molecular weight of the dialysis bags is 3500.

The instruments for characterizations. Diameter and diameter distribution of the nanoparticles were determined by Malvern Zeta-sizer Nano for dynamic light scattering (DLS). The measurement was carried out at 25 ^{o}C and the scattering angle was fixed at 90°. Transmission electron microscopy (TEM) images were taken by a JEOL JEM-1011 (Japan) at the accelerating voltage of 100 kV. To prepare specimens for TEM, a drop of NPs solution (0.1 mg/mL) was deposited onto a copper grid with a carbon coating. The specimens were air-dried and measured at room temperature. UV−vis absorption spectra were recorded via a Shimadzu UV-2450 UV−vis scanning spectrophotometer. Fluorescence emission spectra were conducted on a LS-55

fluorophotometer. Fluorescence quantum efficiency was obtained on a Hamamatsu Absolute PL Quantum Yield Measurement System C9920-02. The cell confocal images were obtained using Zeiss confocal laser microscope (ZEISS LSM 700). Flow cytometry was carried out on Guava easyCyte 6-2L Base System (Merck Millipore, USA).

Measurement of fluorescence quantum yield. We firstly detected the concentration of DSA in DPP NRs/DPP NSs/DSA NSs by absorbance curve, respectively. Then we adjusted them with the same concentration of DSA and also prepared DSA THF solution with the same concentration. Fluorescence quantum efficiency was obtained on a Hamamatsu Absolute PL Quantum Yield Measurement System C9920-02.

Cell culture. HeLa cells were purchased from the Institute of Biochemistry and Cell Biology, Chinese Academy of Sciences, Shanghai, China. The cells were propagated to confluence in Dulbecco's modified Eagle's medium (DMEM, GIBCO) supplemented with 100 U/mL penicillin, 100 μg/mL streptomycin (Sigma) and heat-inactivated fetal bovine serum (FBS, GIBCO), and maintained at 37°C in a humidified atmosphere of 5% CO_2 for further cell experiments.

Biocompatibility of polymer, DSA, DPP NRs/DPP NSs/DSA NSs in vitro by MTT Assay. Cells harvested in a logarithmic growth phase were seeded in 96-well plates at a density of 8×10^3 cells per well and incubated in DMEM for 24 h. The medium was then replaced by 200 μL of DMEM containing predetermined concentrations of various polymer, and then incubated for 24 h, followed by MTT assays to measure the live cells. Cell viabilities were determined by reading the absorbance of the plates at 490 nm with a microplate reader. The cells incubated with DMEM were used as the control. The cell viability (%) =A sample /A control ×100%. We also detected the cytotoxicity of various polymer for different culture time. The procedures were the same for that of DPP NRs/DPP NSs/DSA NSs.

Live/ dead cell staining assays. To further confirm the toxicity of DPP NRs/DPP NSs/DSA NSs, HeLa cells were stained with the calcein-AM/propidium iodide (PI) to determine their viabilities. Briefly, the cells were incubated with DPP NRs/DPP NSs/DSA NSs, for 24 h. The cells without any treatment were set as the controls.

After that, the cells were further incubated at 37°C for additional 24 h. The cells were stained with calcein AM (green for living cells) and PI (red for dead cells) for 30 min at room temperature, and imaged with a confocal fluorescence microscope.

Cellular uptake and tracking in vitro. The Cellular uptake of nanoparticles was examined by using a confocal laser scanning microscope (CLSM). Cells harvested in a logarithmic growth phase were seeded in 6-well plates (a sterile cover slip was put in each well) at a density of 2.5×10^{5} cells/well and incubated in DMEM for 24 h. The medium was then replaced by 2 mL of DMEM containing nanoparticles and incubated for different hours at 37°C, and further washed using PBS for 3 times.

For the CLSM detection, the cells were fixed with 4% of paraformaldehyde solution for 10 min. After that, DAPI (4,6-diamidino-2-phenylindole) was added for another 5 min incubation to locate the nucleus. Later, the cells were washed with PBS and observed using confocal laser scanning microscopy (CLSM, Zeiss LSM 700).

For the flow cytometry detection, the cells were washed with PBS and treated with trypsin. The harvested cells were suspended in PBS and centrifuged at 1000 rpm for 5 min. The supernatants were discarded and the cells were washed again with PBS to remove the medium. After washing, the cells were re-suspended in 500 μL PBS before analysis by Guava easyCyte 6-2L Base System (Merck Millipore, USA).

Endocytosis pathway detection. Cells harvested in a logarithmic growth phase were seeded in 6-well plates at a density of 2.5×10^{5} cells/well and incubated in DMEM for 24 h. Then, serum-free DMEM as the control and various inhibitors including sucrose (clathrin-mediated endocytosis, 450 mM), genistein (caveolin-dependent endocytosis, 100 μM), Amiloride (micropinocytosis, 13.3 μg mL^{-1}) were used in serum-free DMEM for 1 h and 4°C culturing was used to inhibit energy-dependent mechanisms. Then, DPP NRs/DPP NSs/DSA NSs were further added for 4 h incubation, respectively. Subsequently, the cells were collected and re-suspended in 0.5 mL PBS 7.4. Flow cytometry analysis was performed by Guava easyCyte 6-2L Base System (Merck Millipore, USA) which collected 1×10^{4} gated events for each sample.

Long-term cellular imaging. Cells harvested in a logarithmic growth phase were seeded in 6-well plates at a density of 2.5×10^{5} cells/well and incubated in DMEM for

24 h. The medium was then replaced by 2 mL of DMEM containing DPP NRs/DPP NSs/DSA NSs with 8 μg mL^{-1} of DSA and incubated for 6 hours at 37°C (Day 0). Then the cells were diluted and subcultured in 6-well plates containing cell culture coverslips for designated days (from day 0 to day 15). Upon reaching designated day, the cells were washed with PBS buffer and then fixed with 4% of paraformaldehyde solution for 10 min. Later, the cells were washed with PBS and observed using confocal laser scanning microscopy (CLSM, Zeiss LSM 700).

Animal experiments. All animal experiments were performed complying with the NIH guidelines for the care and use of laboratory animals. HeLa cells were administered by subcutaneous injection into the right flank region of the male BALB/c mice.

In vivo imaging in living mice. We choose the mice bear the tumor on the right flank to carry out this study. In order to detect the imaging capacity, DPP NRs and DPP NSs (1 mg/kg DSA) were administrated into the mice via intratumor or the tail vein injection. Then, under anesthesia, the in vivo imaging was performed using an in vivo imaging system at different time periods post-injection, respectively. Maestro software was used to remove the mouse background fluorescence.

In vivo biodistribution analysis. Mice bearing tumors were randomly assigned into two groups when the tumor volumes reached 100 – 200mm^3. All the mice were injected via the tail vein with 1 mg/kg DSA. At 48 h after injection, the heart, liver, spleen, lungs, kidneys, and tumors were collected for imaging and semi-quantitative biodistribution analysis.

In vivo biocompatibility assessment. The female BALB/c nude mice (18~22 g) were intravenously injected with saline,PEG-PLA, PEG-PCL, DPP NRs or DPP NSs at DSA dose of 1 mg/kg or polymer dose of 20 mg/kg, respectively. After 48 hours, whole blood and serum were collected and followed by sacrificing the mice. The blood samples were used to detect alanine aminotransferase (ALT), aspartate aminotransferase (AST), blood urea nitrogen (BUN) and creatinine (CREA) parameters for the biochemical analysis.

2. Figures and Tables

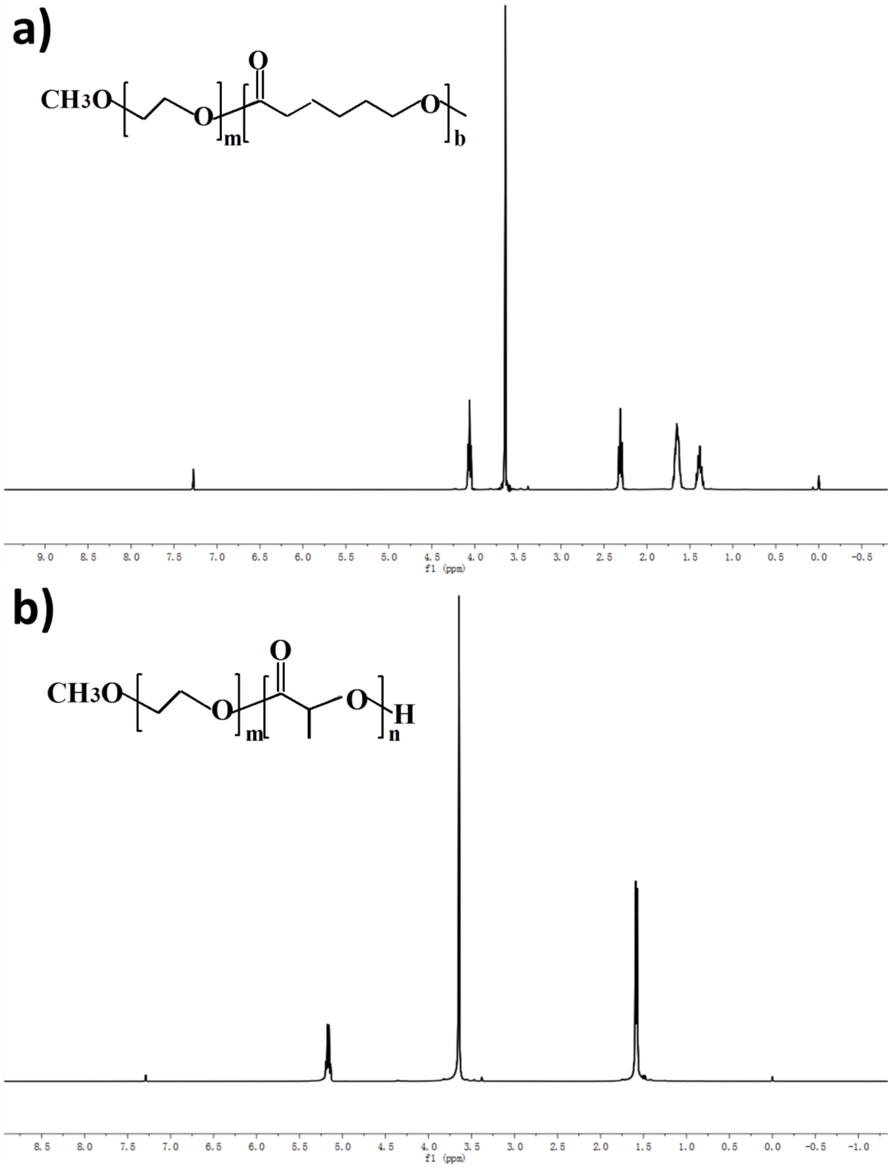

Fig. S1. ^{1}H NMR spectrum of a) PEG_{5k}-PCL_{10k} and b) PEG_{5k}-PLA_{10k} in $CDCl_3$.

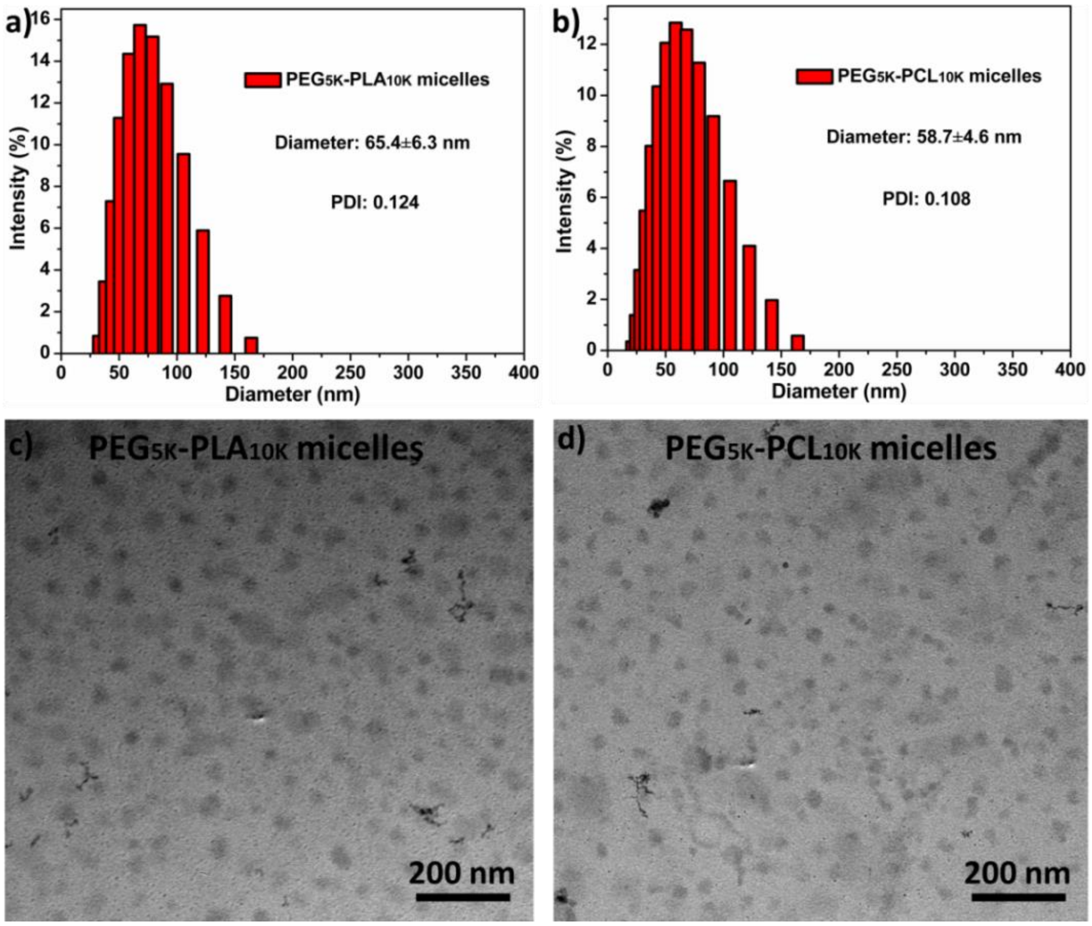

Fig. S2. Size distribution of PEG_{5K}-PLA_{10K} micelles a) and PEG_{5K}-PCL_{10K} micelles b), respectively. TEM images of PEG_{5K}-PLA_{10K} micelles c) and PEG_{5K}-PCL_{10K} micelles d), respectively. Scale bars are 200 nm.

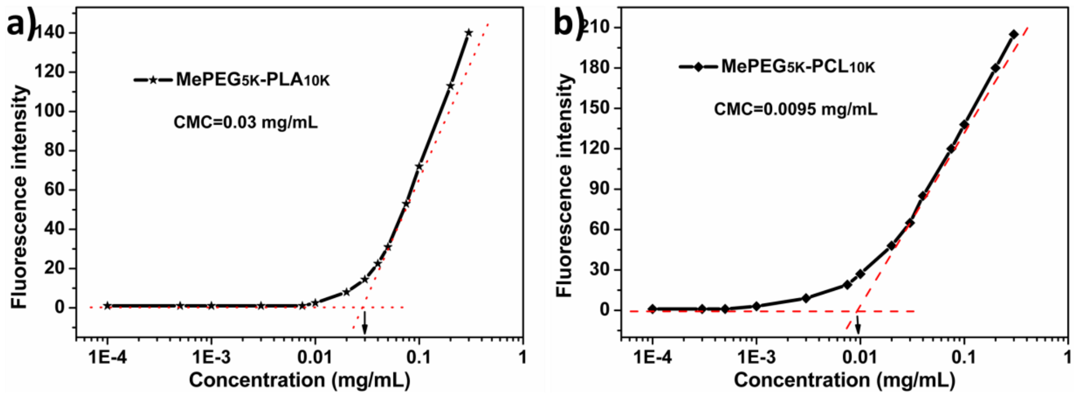

Fig. S3. Fluorescence intensity of Nile red from the fluorescence spectra with a) PEG_{5K}-PLA_{10K} micelles or b) PEG_{5K}-PCL_{10K} micelles in different concentration.

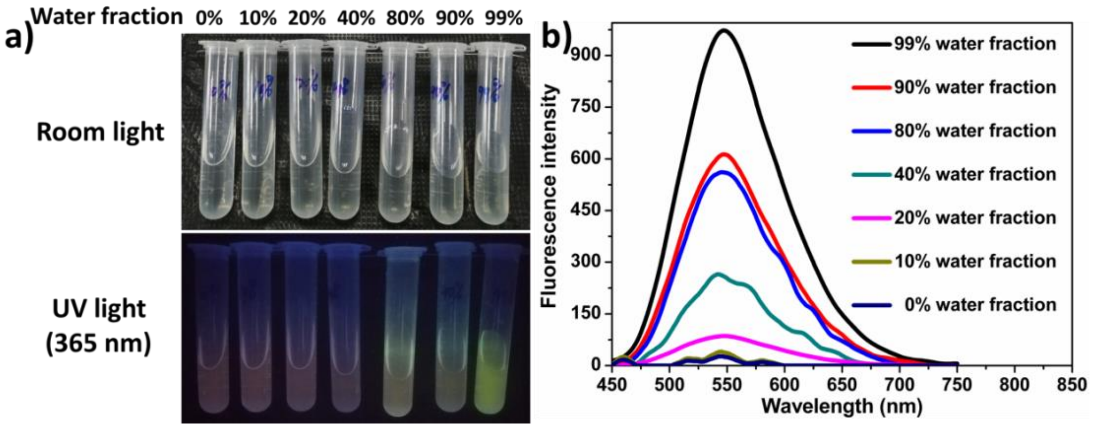

Fig. S4. a) Photographs of DSA in THF/water mixtures with different water volume fractions under room light (upper picture) and UV light (lower picture), respectively. b) Fluorescence spectra of DSA in THF/water mixtures with different water volume fractions (0, 10, 20, 40, 80, 90 and 99 vol %), respectively.

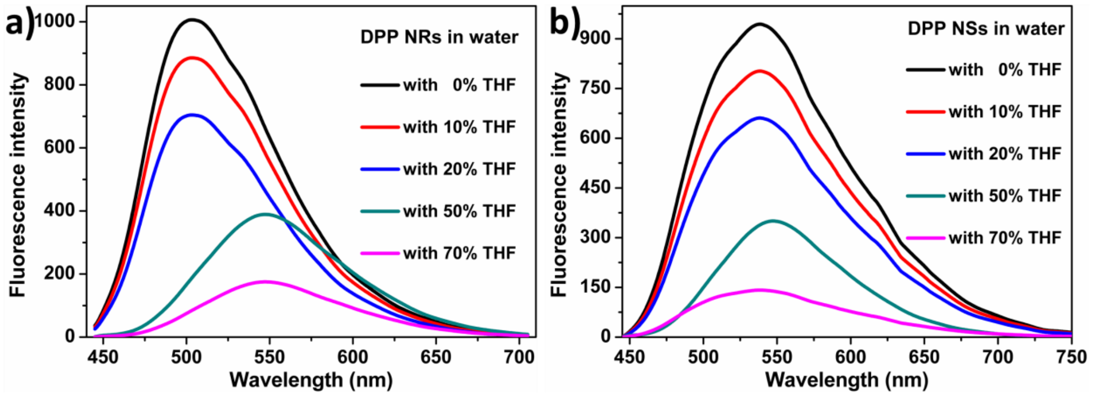

Fig. S5. Fluorescence spectra of a) DPP NRs and b) in THF/water mixtures with different THF volume fractions (0, 10, 20, 50 and 70 vol %), respectively.

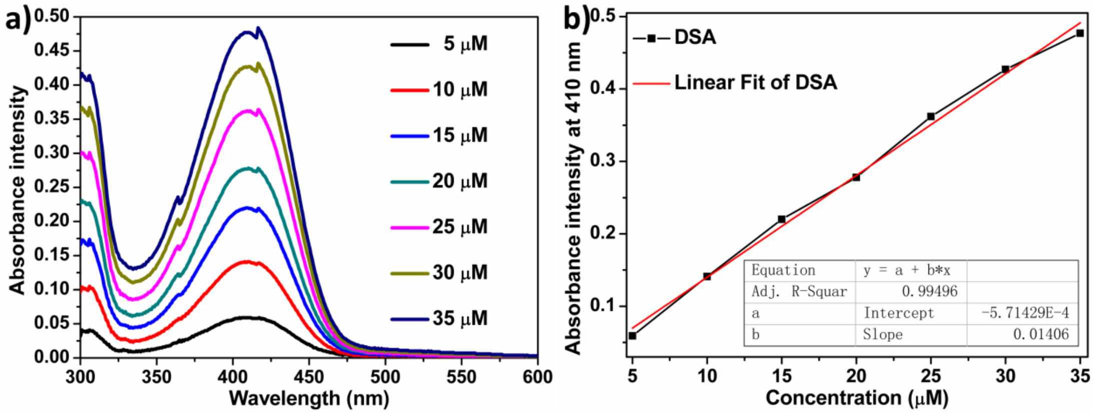

Fig. S6. a) and b) Standard absorbance curve of DSA. (The absorbance of DSA molecules at 410 nm (from a mixture of THF and water (v/v = 4:1)) as a function of DSA concentration.)

Table S1. Characterizations of DPP NRs, DPP NSs and DSA NSs, respectively.

Morphology	Average size (nm)	PDI	EE%	Quantum yield (ø)	Fluorescence lifetime (ns)
DPP NRs	137.1±12.8	0.216	61.2±3.16	58.67%	2.02
DPP NSs	85.3±13.2	0.152	49.5±6.24	67.71%	1.26
DSA NSs	235.4±21.5	0.234	85.4±4.38	60.39%	1.66
DSA				28.41%	2.71

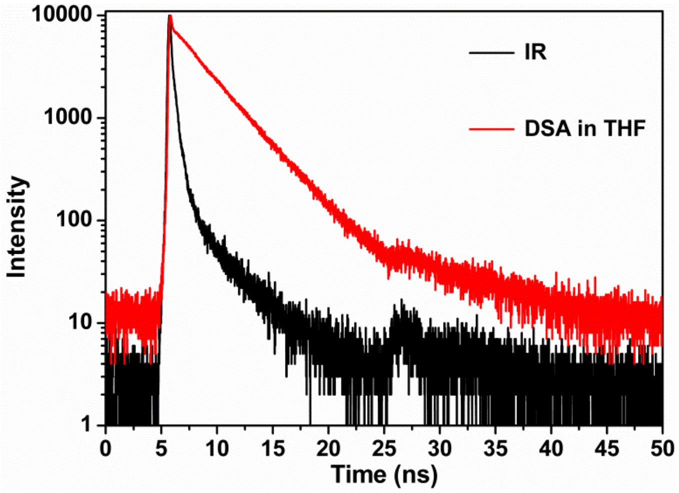

Fig. S7. Time-resolved decay profiles of DSA in THF.

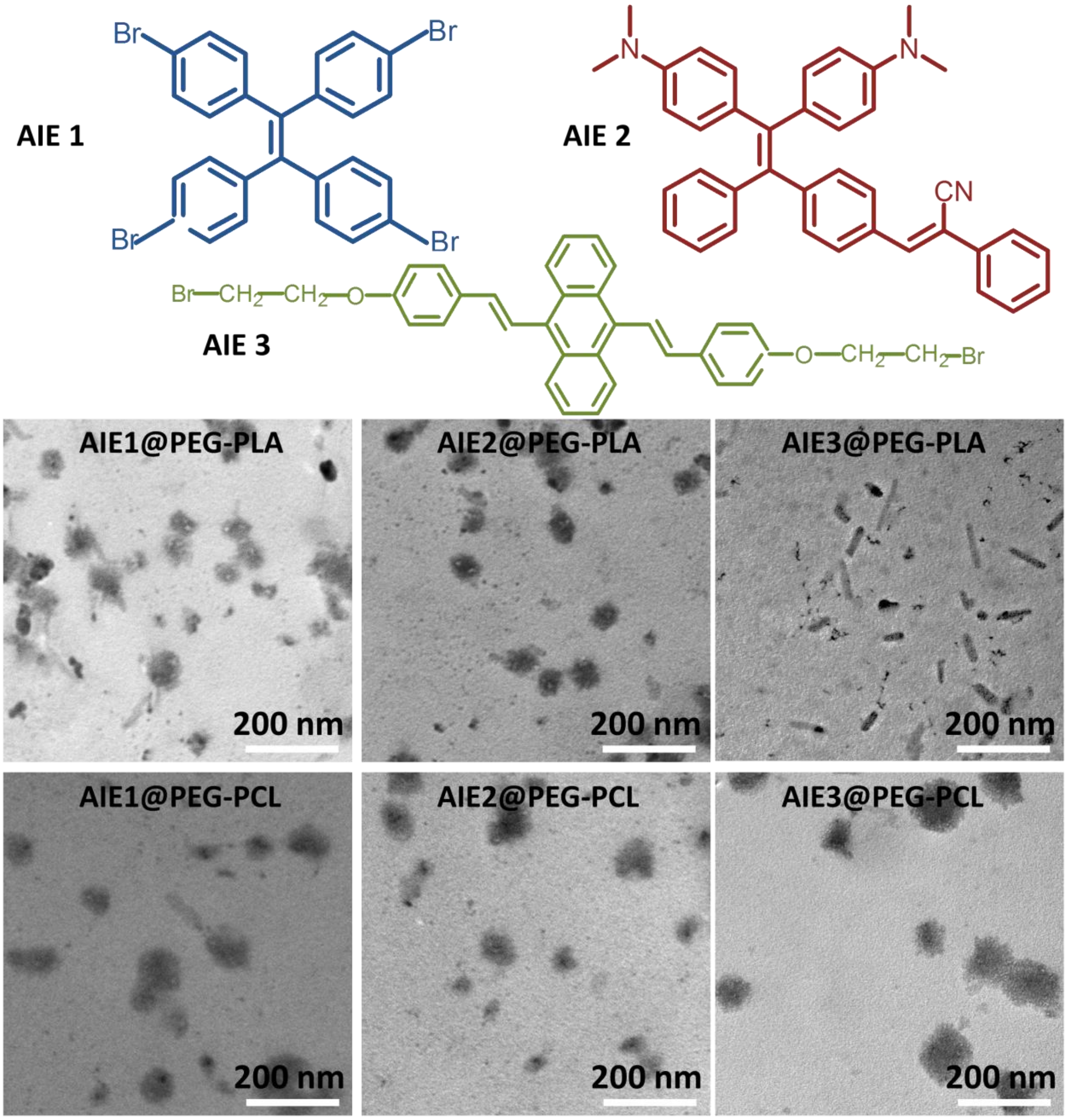

Fig. S8. TEM images of nanoparticles prepared by different AIEgens encapsulated by PEG_{5K}-PLA_{10K} or PEG_{5K}-PCL_{10K}, respectively. Scale bars are 200 nm.

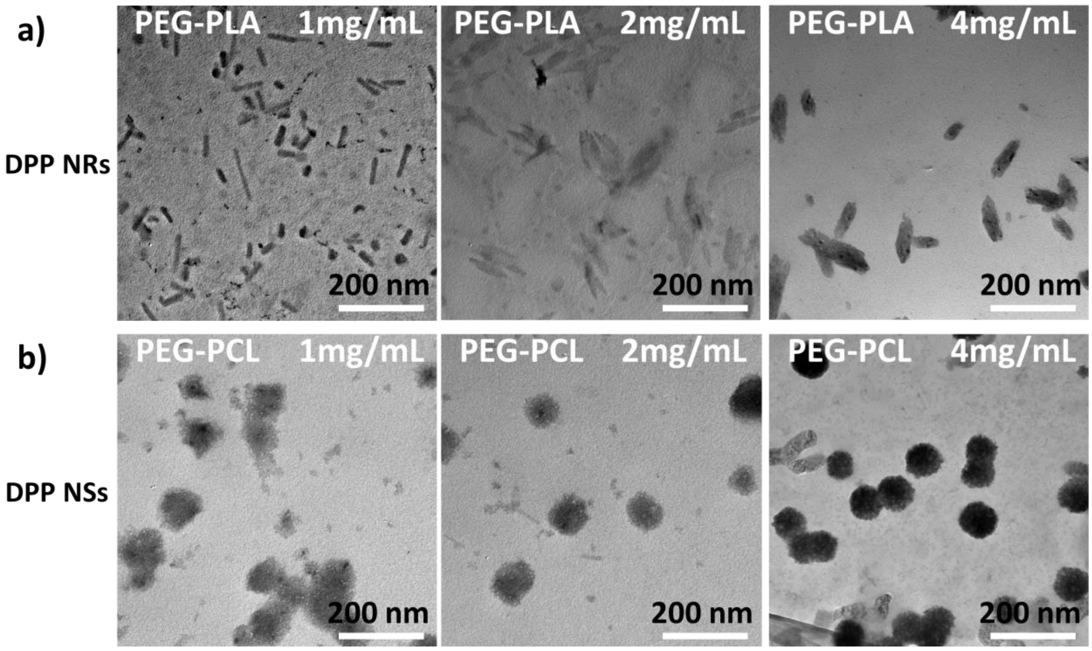

Fig. S9. TEM images of a) DPP NRs and b) DPP NSs prepared with different concentrations of copolymer, respectively. Scale bars are 200 nm.

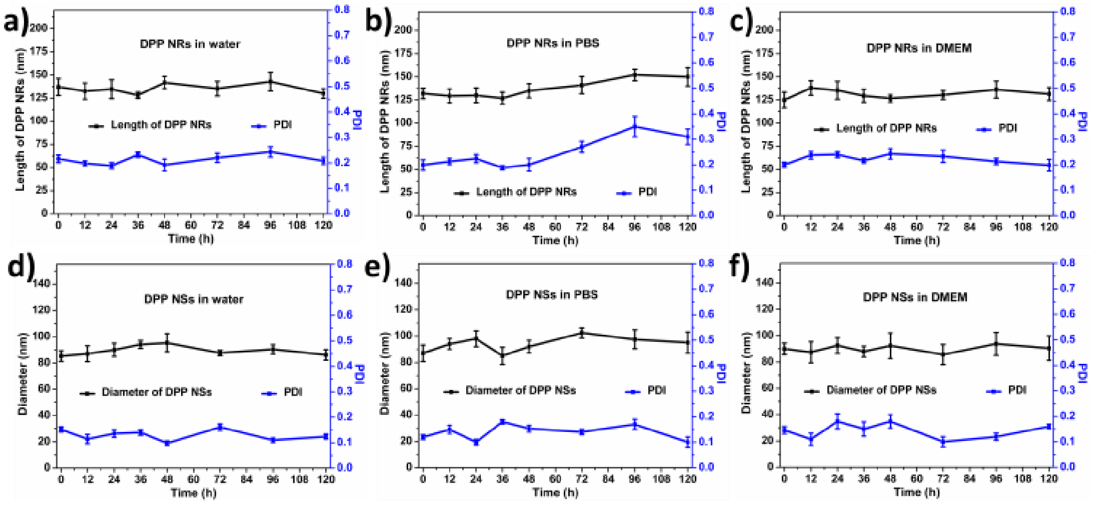

Fig. S10. Average size changes of DPP NRs (a-c) and DPP NSs (d-f) over 120 h in different solutions. The data are shown as the mean values ± standard deviation (SD) (n=3).

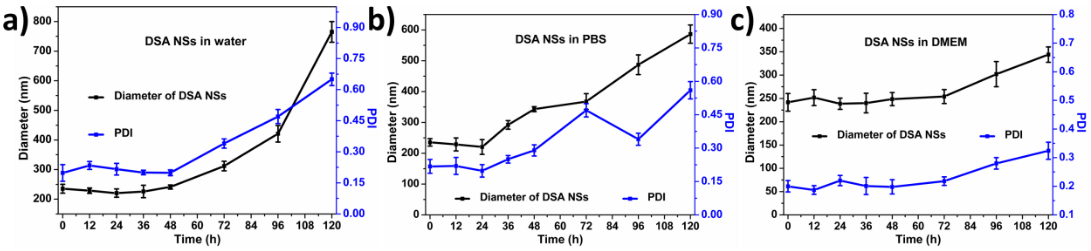

Fig. S11. Average size changes of DSA NSs (a-c) over 120 h in different solutions. The data are shown as the mean values ± standard deviation (SD) (n=3).

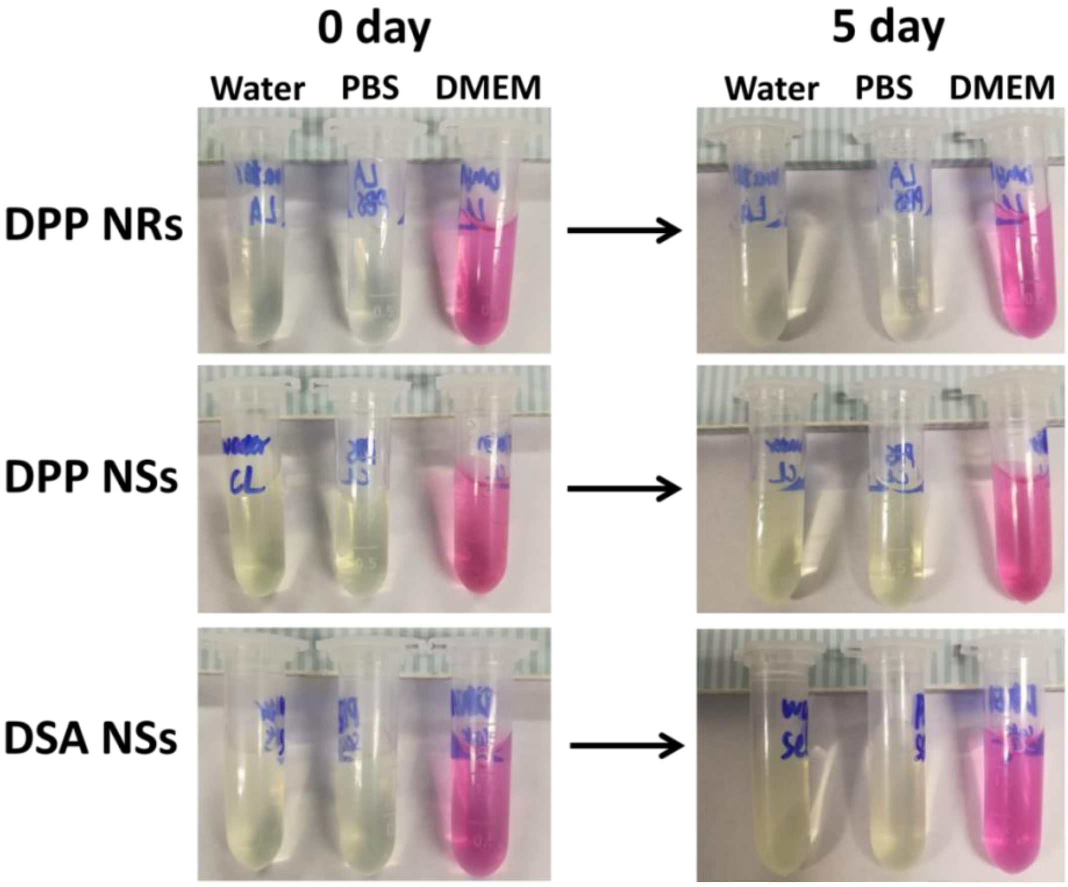

Fig. S12. Photographs of DPP NRs, DPP NSs and DSA NSs under different conditions at 0 day and 5 day, respectively.

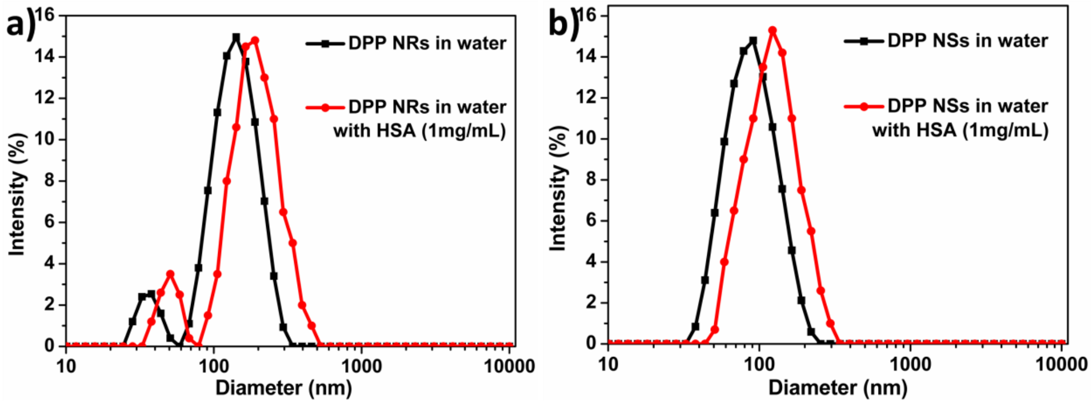

Fig. S13. Size distribution of a) DPP NRs and b) DPP NSs before or after treated with 1 mg/mL of HSA in water.

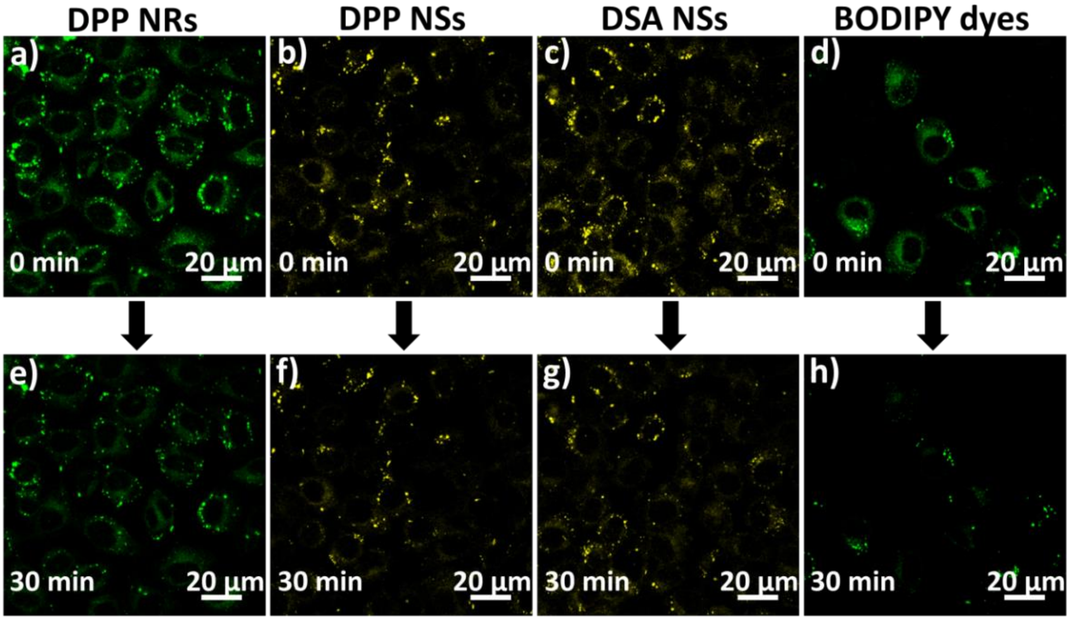

Fig. S14. The confocal images of the HeLa cells stained by DPP NRs (a, e), DPP NSs (b, f), DSA NSs (c, g) and BODIPY dyes (d, h) before (0 min) and after the laser irradiation for 30 min, respectively.

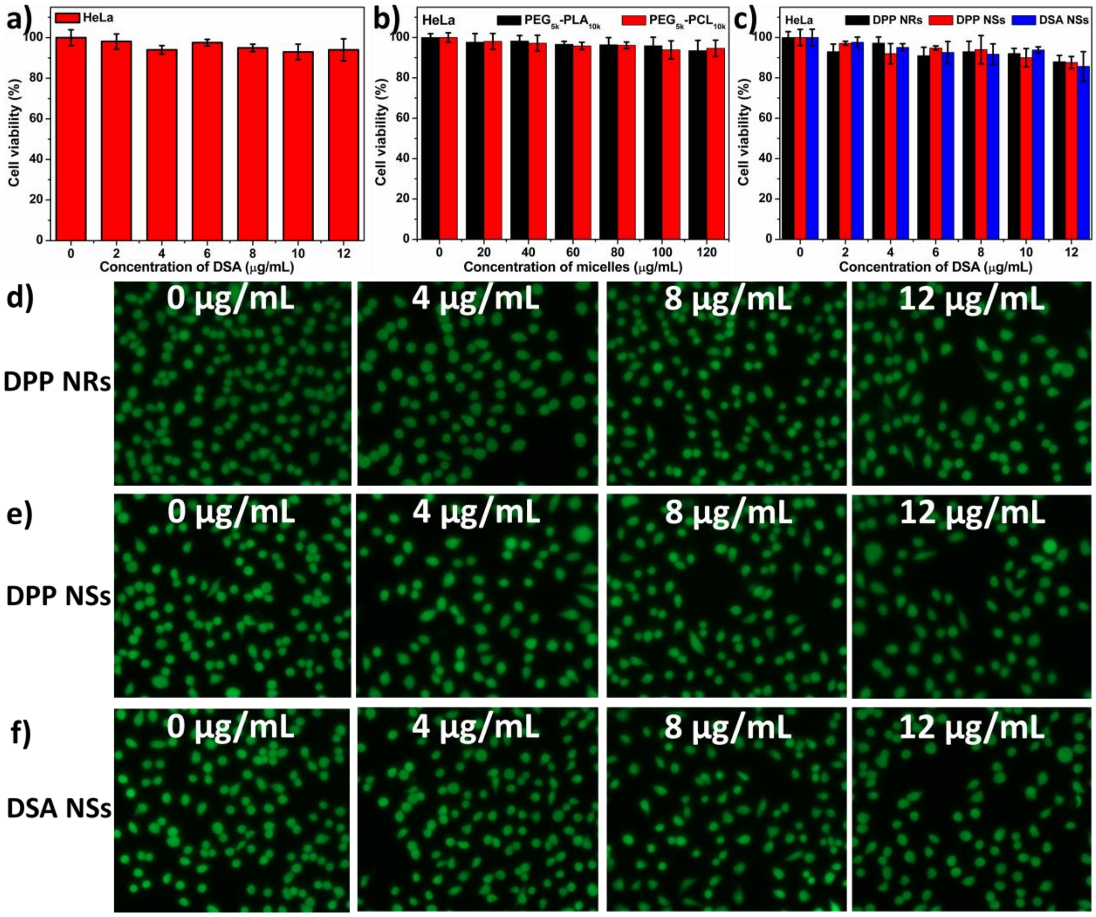

Fig. S15. a) Cell viability of HeLa cells after incubation with various concentrations of DSA for 24 h. b) Cell viability of HeLa cells after incubation with various concentrations of PEG_{5K}-PLA_{10K} and PEG_{5K}-PCL_{10K} for 24 h, respectively. c) Cell viability of HeLa cells after incubation with various concentrations of DPP NRs, DPP NSs and DSA NSs for 24 h, respectively. Data represent mean values ± standard deviation, n=3. d-f) Fluorescence images of HeLa cells after incubation with various concentrations of DPP NRs, DPP NSs and DSA NSs for 24 h, respectively. Green calcein fluorescence indicated live cells.

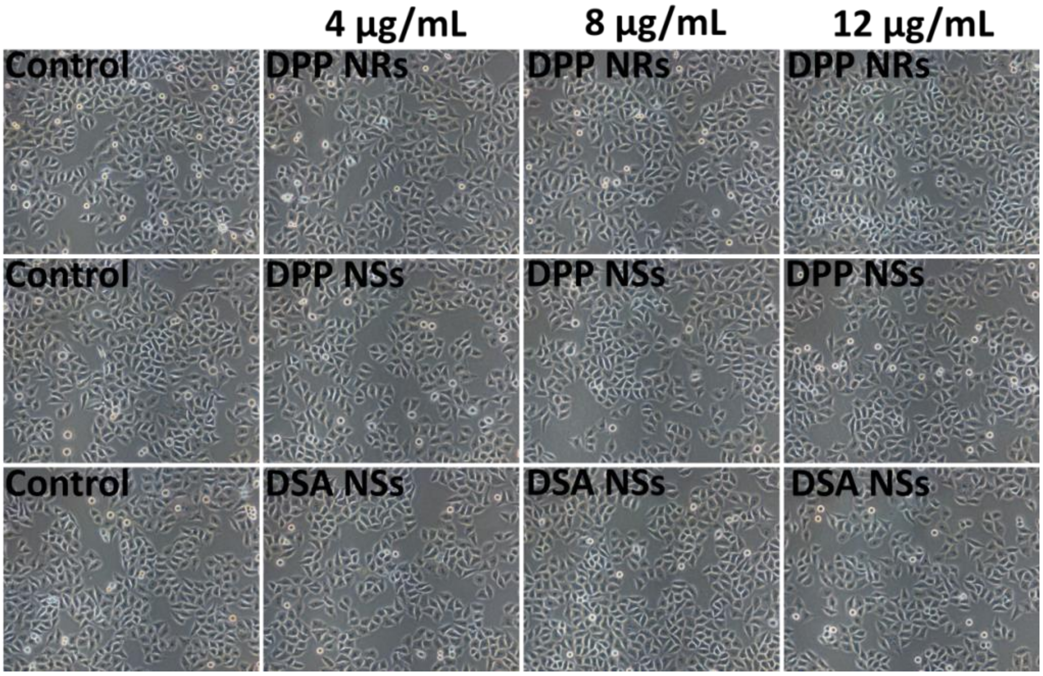

Fig. S16. The morphology change of HeLa cells incubated with DPP NRs, DPP NSs or DSA NSs of different concentration of DSA for 24 h at 37 ^{o}C.

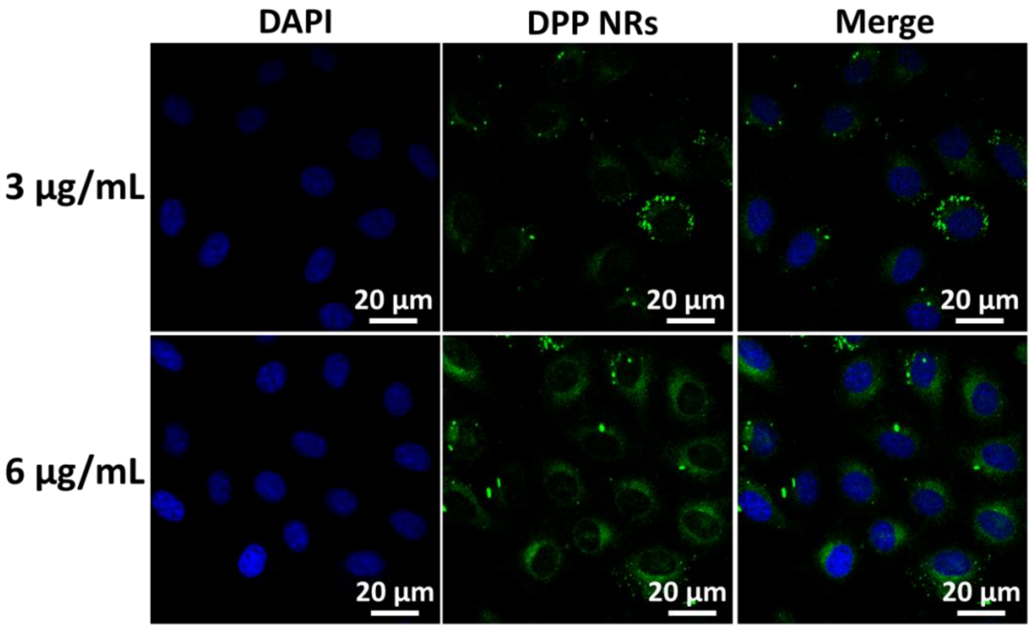

Fig. S17. CLSM images showing changes in the signal of DSA in HeLa cells treated with DPP NRs with the DSA concentration of 3 μg/mL (upper) and 6 μg/mL (lower) for 2 h at 37 ^{o}C. Scale bars: 20 μm.

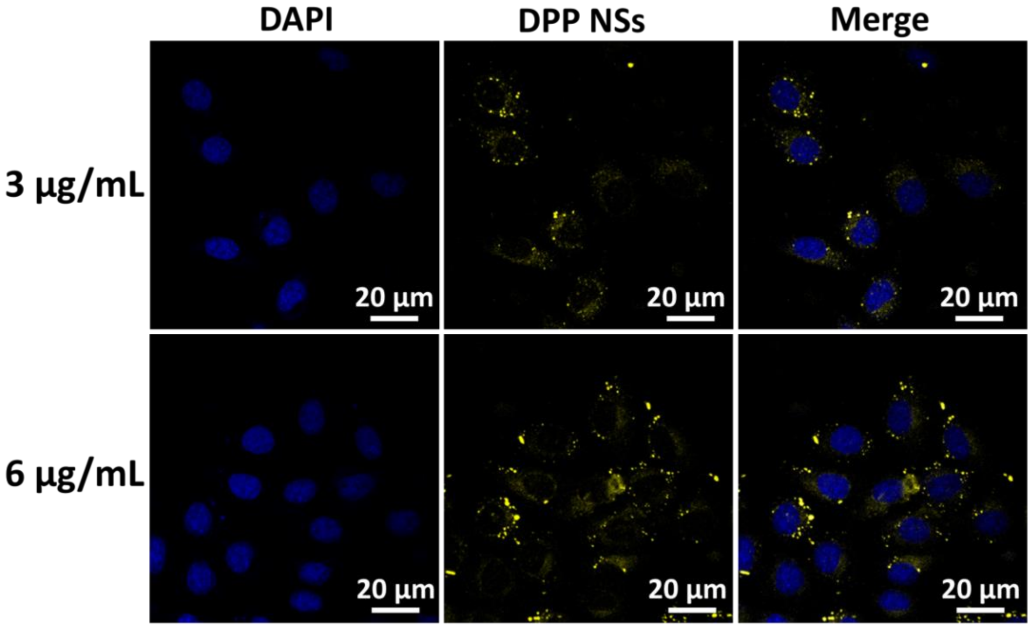

Fig. S18. CLSM images showing changes in the signal of DSA in HeLa cells treated with DPP NSs with the DSA concentration of 3 µg/mL (upper) and 6 µg/mL (lower) for 2 h at 37 ^{o}C. Scale bars: 20 µm.

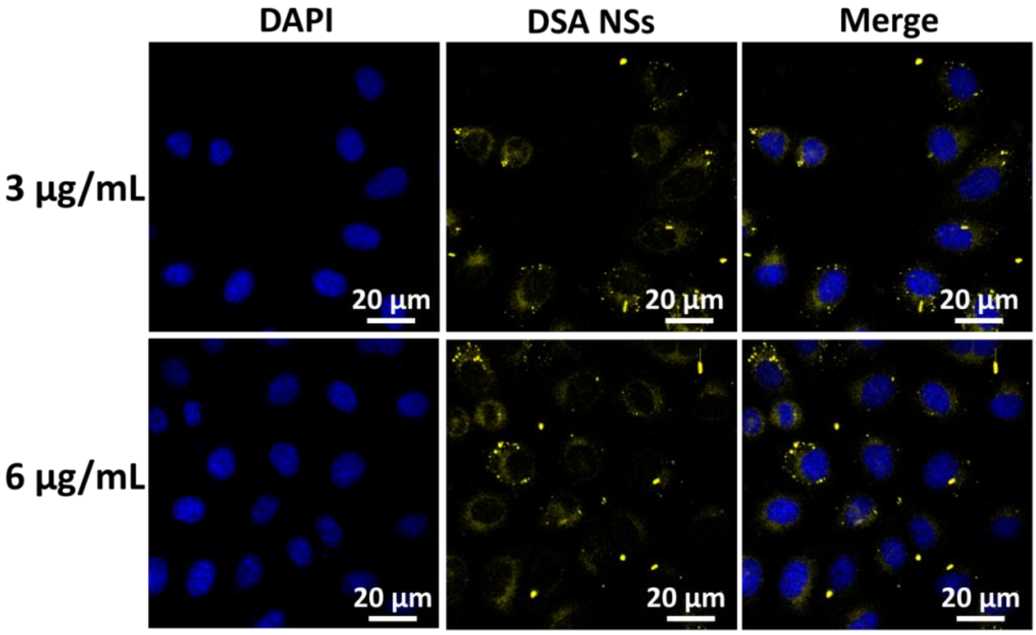

Fig. S19. CLSM images showing changes in the signal of DSA in HeLa cells treated with DSA NSs with the DSA concentration of 3 µg/mL (upper) and 6 µg/mL (lower) for 2 h at 37 ^{o}C. Scale bars: 20 µm.

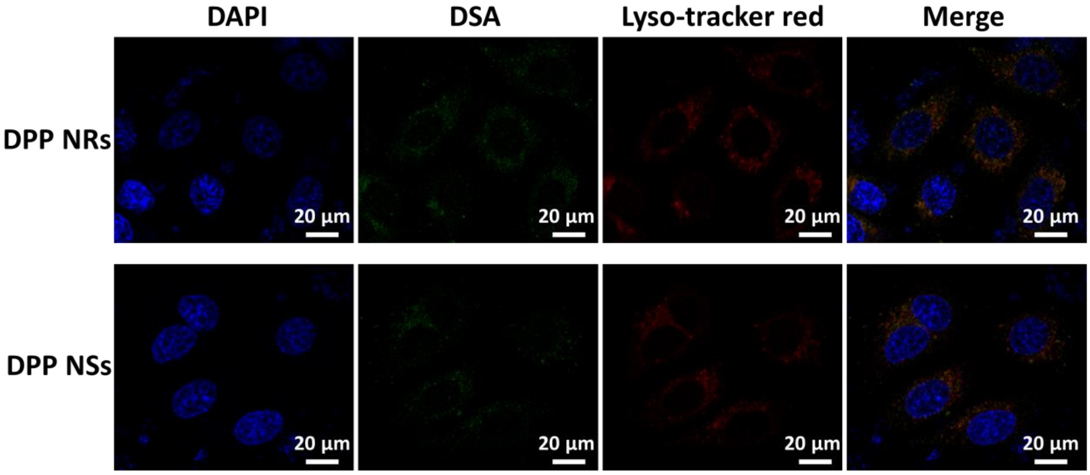

Fig. S20. Intracellular uptake of DPP NRs or DPP NSs in HeLa cells in the presence of Lyso-tracker Red after incubation for 1 h. Scale bars represent 20 μm in all images.

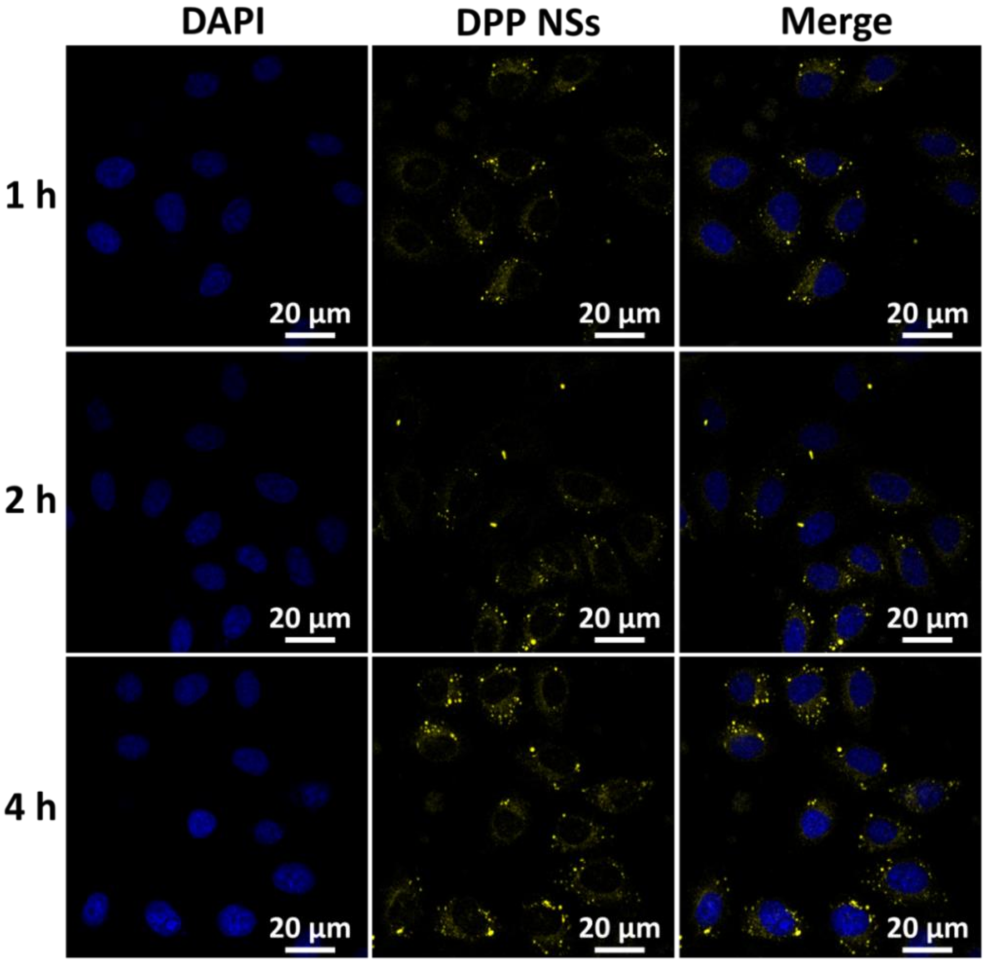

Fig. S21. CLSM images showing changes in the signal of DSA in HeLa cells treated with DPP NSs with the same DSA concentration for 1 h (upper), 2 h (middle) and 4 h (lower) at 37 ^{o}C. Scale bars: 20 μm.

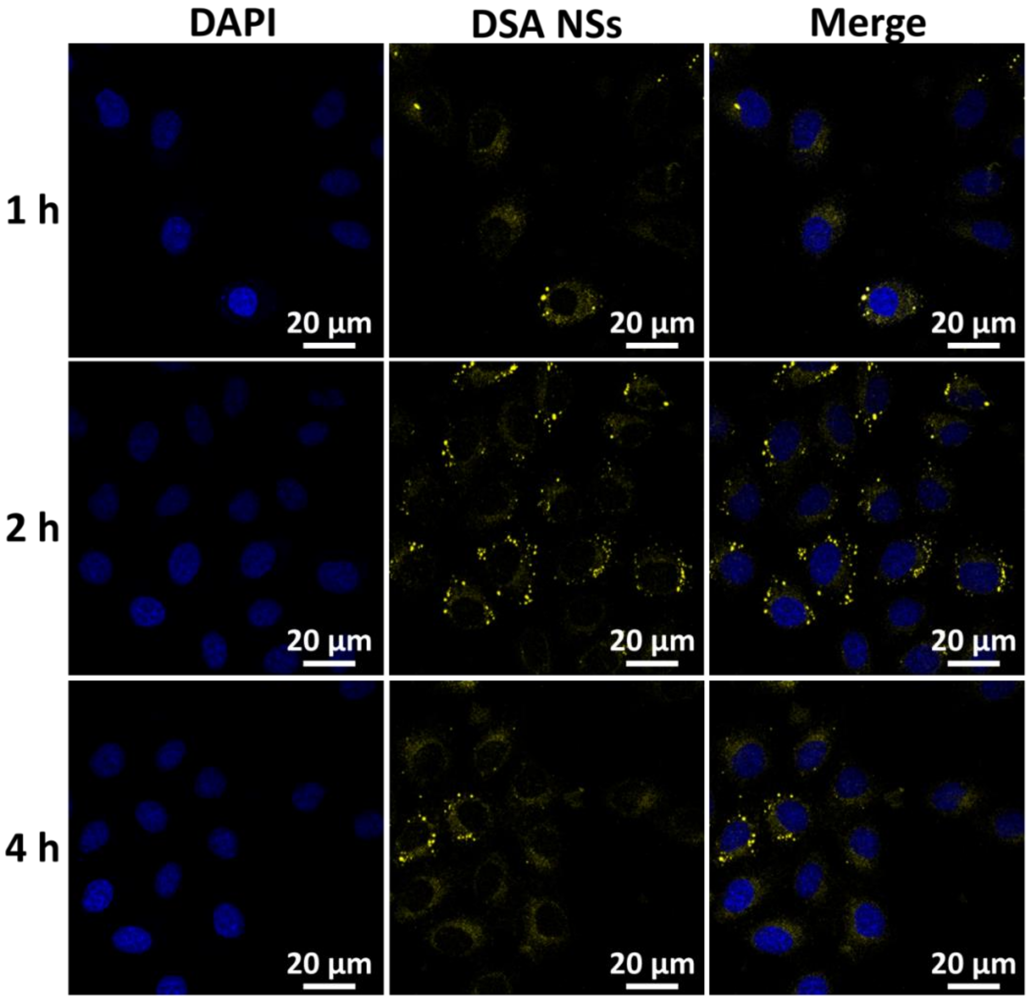

Fig. S22. CLSM images showing changes in the signal of DSA in HeLa cells treated with DSA NSs with the same DSA concentration for 1 h (upper), 2 h (middle) and 4 h (lower) at 37 ^{o}C. Scale bars: 20 µm.

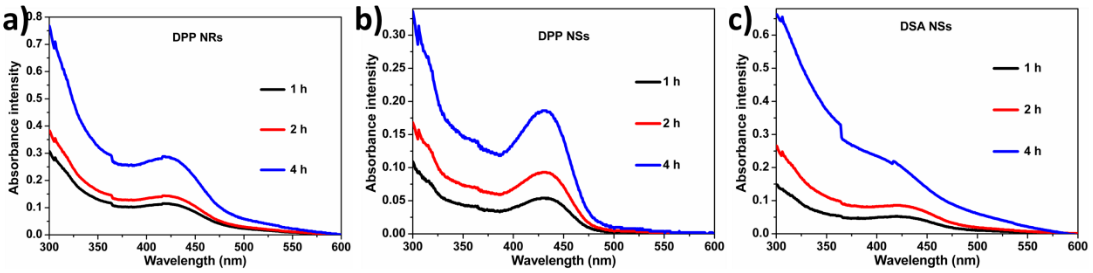

Fig. S23. Absorbance of DSA extracted from HeLa cells after 1, 2, and 4 h incubation with a) DPP NRs, b) DPP NSs and c) DSA NSs, respectively.

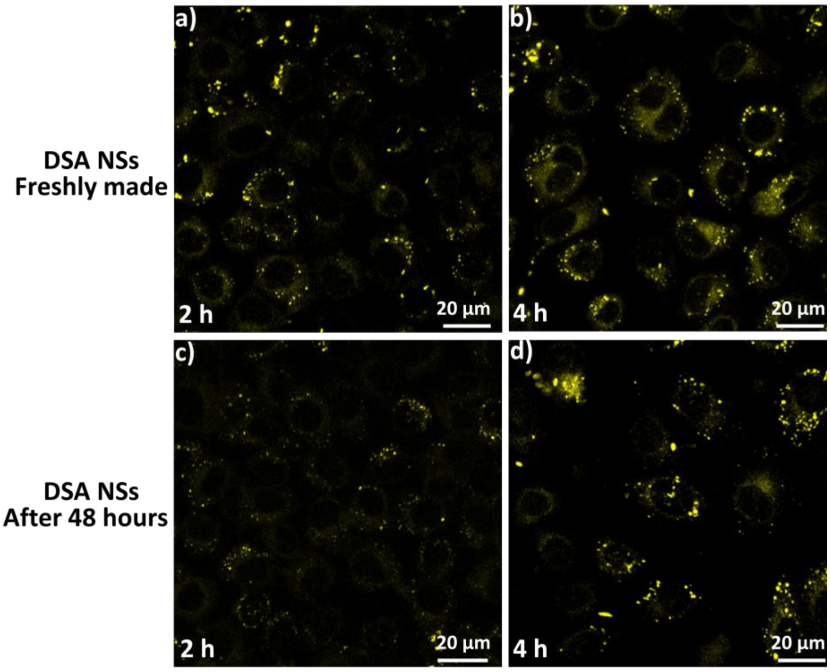

Fig. S24. CLSM images of HeLa cells incubated with DSA NSs (Freshly made) and DSA NSs (After storing 48 hours) for 3 h at 37°C. Scale bars represent 20 µm in all images.

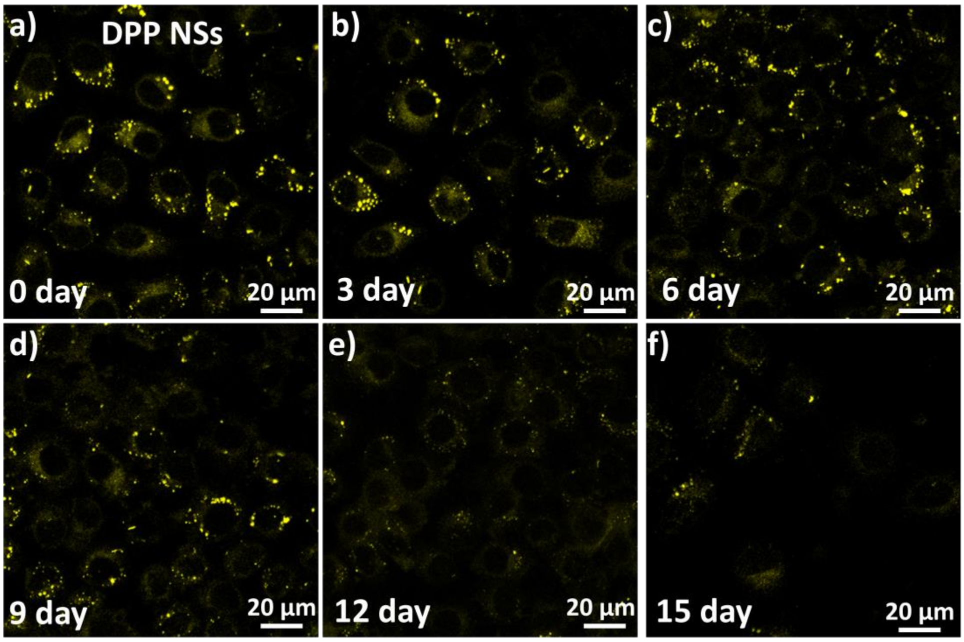

Fig. S25. Long-term cell tracing images of the DPP NSs at 37°C for 6 h and then subcultured for designated time intervals including a) day 0; b) day 3 ; c) day 6; d) day 9; e) day 12; f) day 15. Scale bars represent 20 µm in all images.

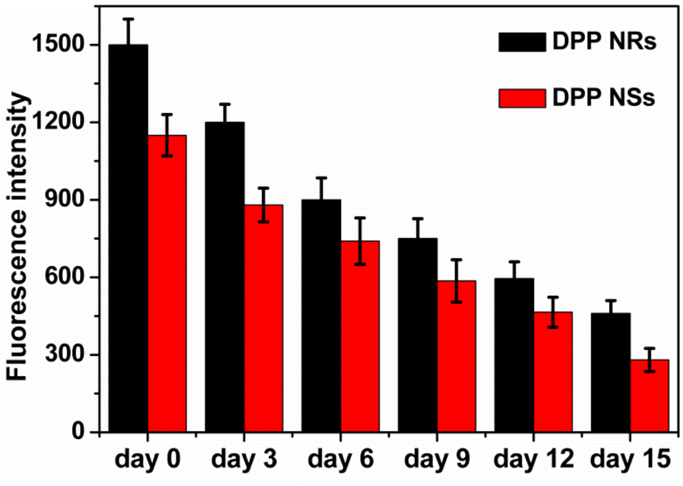

Fig. S26. Time-dependent fluorescence intensity changes for the cells treated with DPP NRs and DPP NSs, respectively.

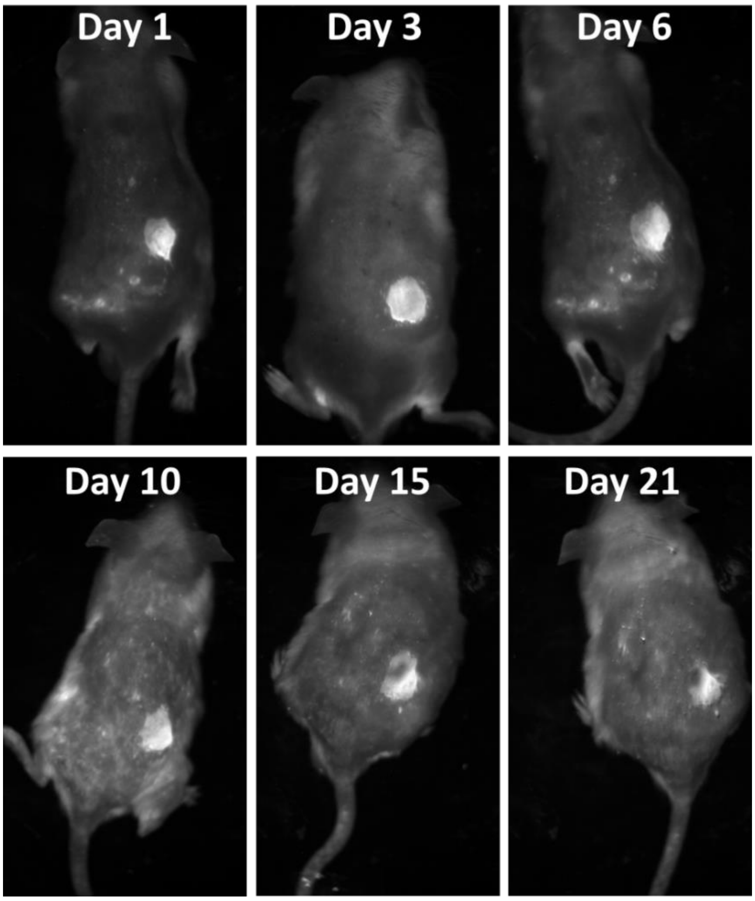

Fig. S27. The gray intensity of mouse injected with DPP NRs during the imaging period.

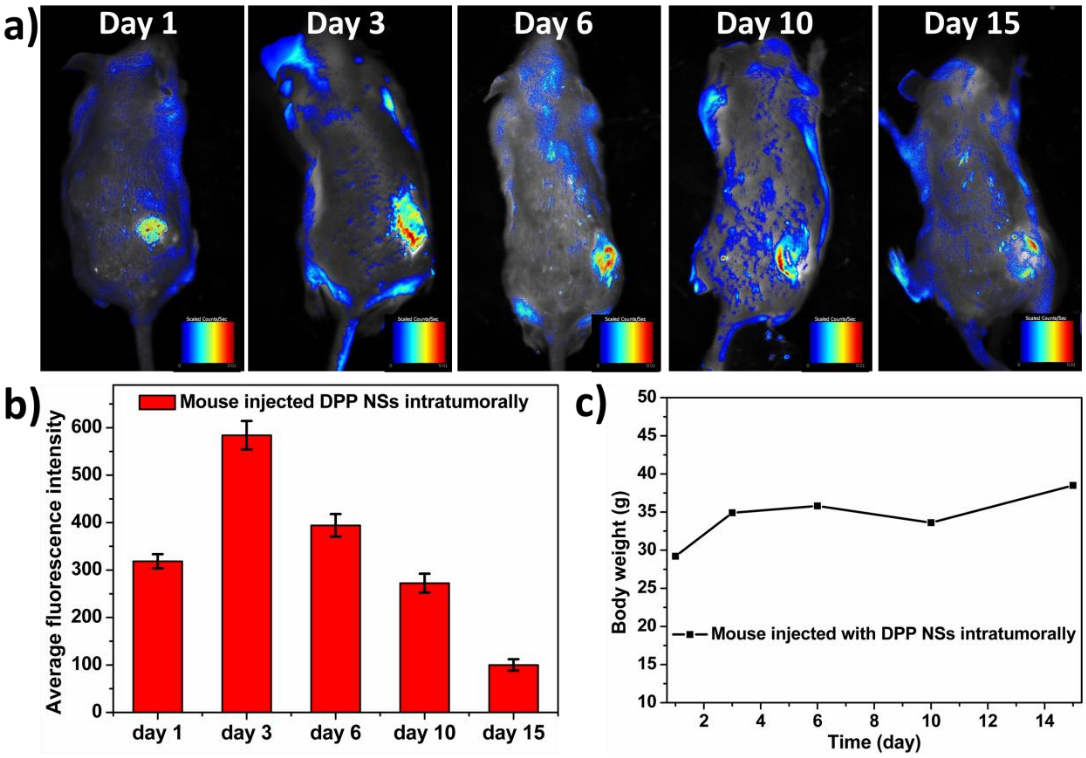

Fig. S28. a) Representative time-dependent *in vivo* fluorescence images of the tumor-bearing mouse that was intratumorally injected with DPP NSs from day 1 to day 15. b) Time-dependent fluorescence intensity changes for the tumors. Data represent mean values ±standard deviation, n=3. c) Body weight of the mouse during the imaging time.

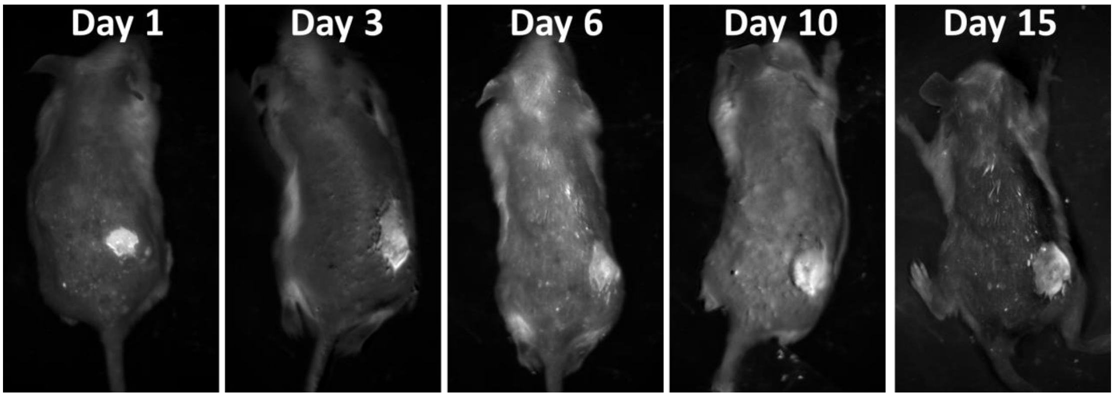

Fig. S29. The gray intensity of mouse injected with DPP NSs during the imaging period.

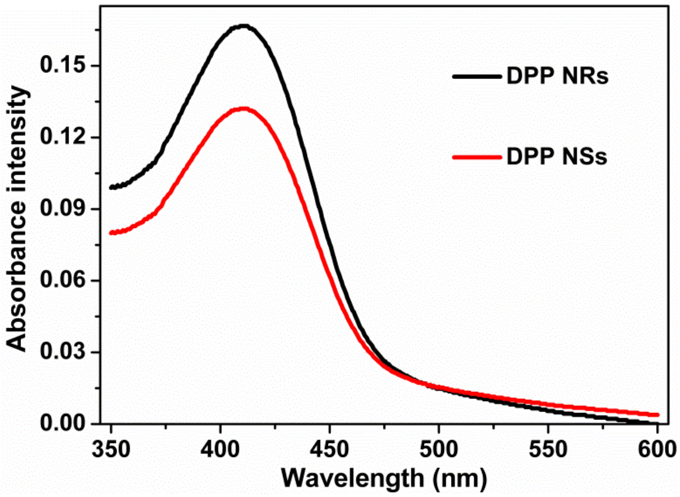

Fig. S30. Absorbance of DSA extracted from tumors after incubation with DPP NRs or DPP NSs for 7 days, respectively.

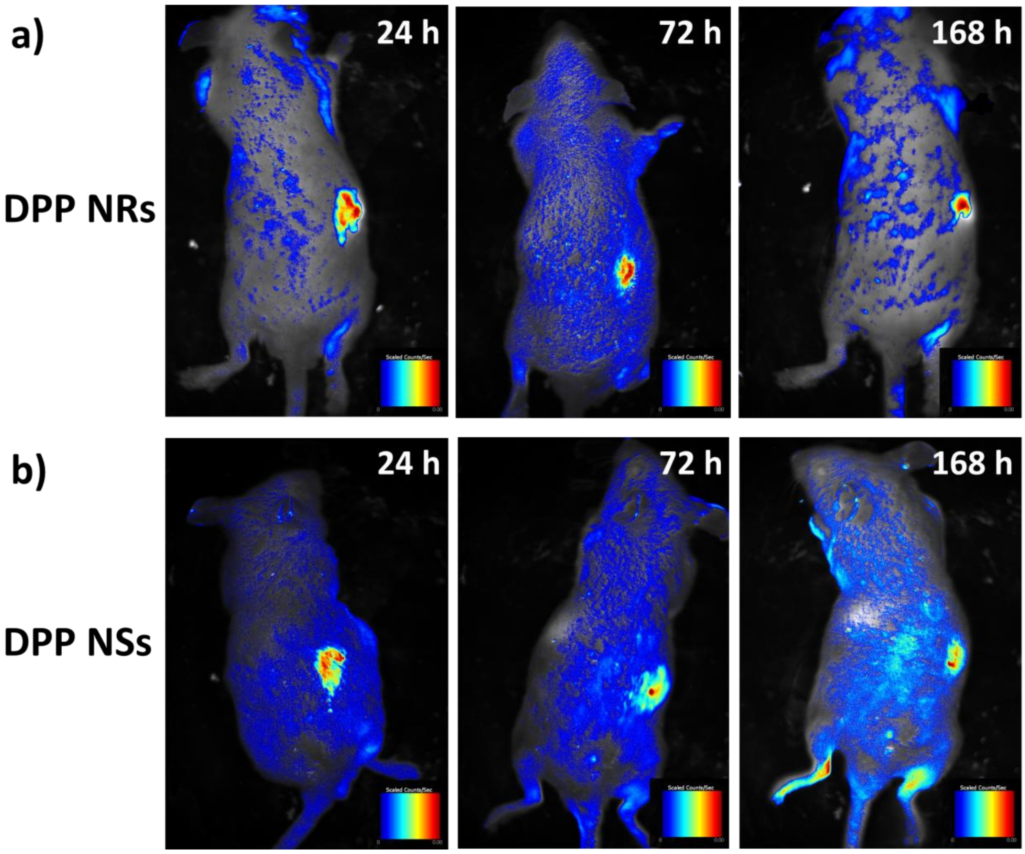

Fig. S31. Representative time-dependent *in vivo* fluorescence images of the tumor-bearing mouse that was intravenously injected with a) DPP NRs or b) DPP NSs from 24 h to 168 h.

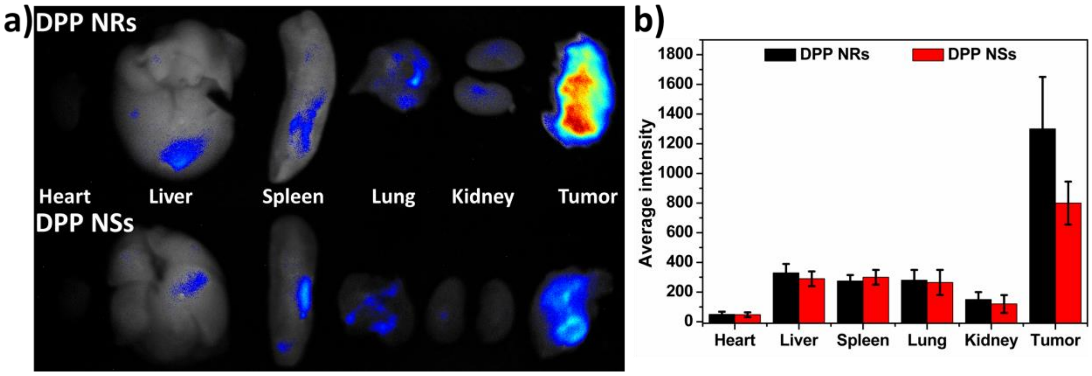

Fig. S32. Fluorescence images of major organs and tumor after intravenous injection of DPP NRs or DPP NSs at 48 h.

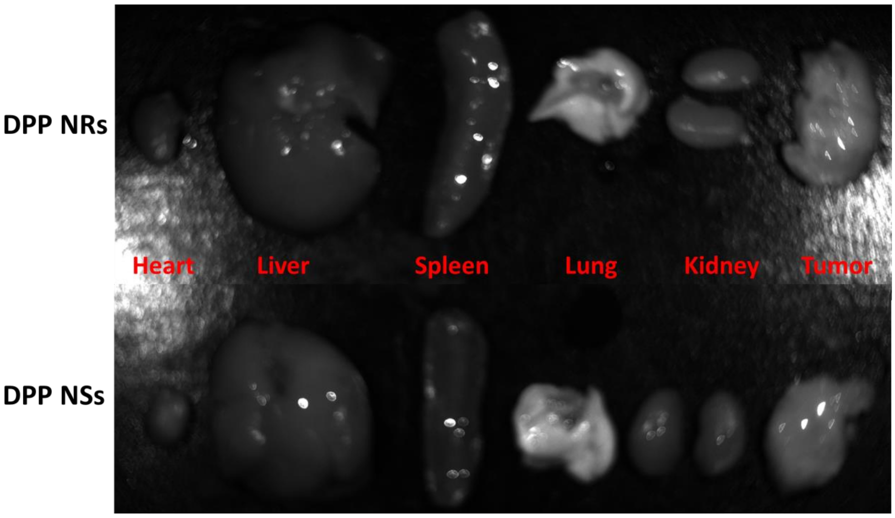

Fig. S33. Photographs of major organs and tumor after intravenous injection of DPP NRs or DPP NSs at 48 h.

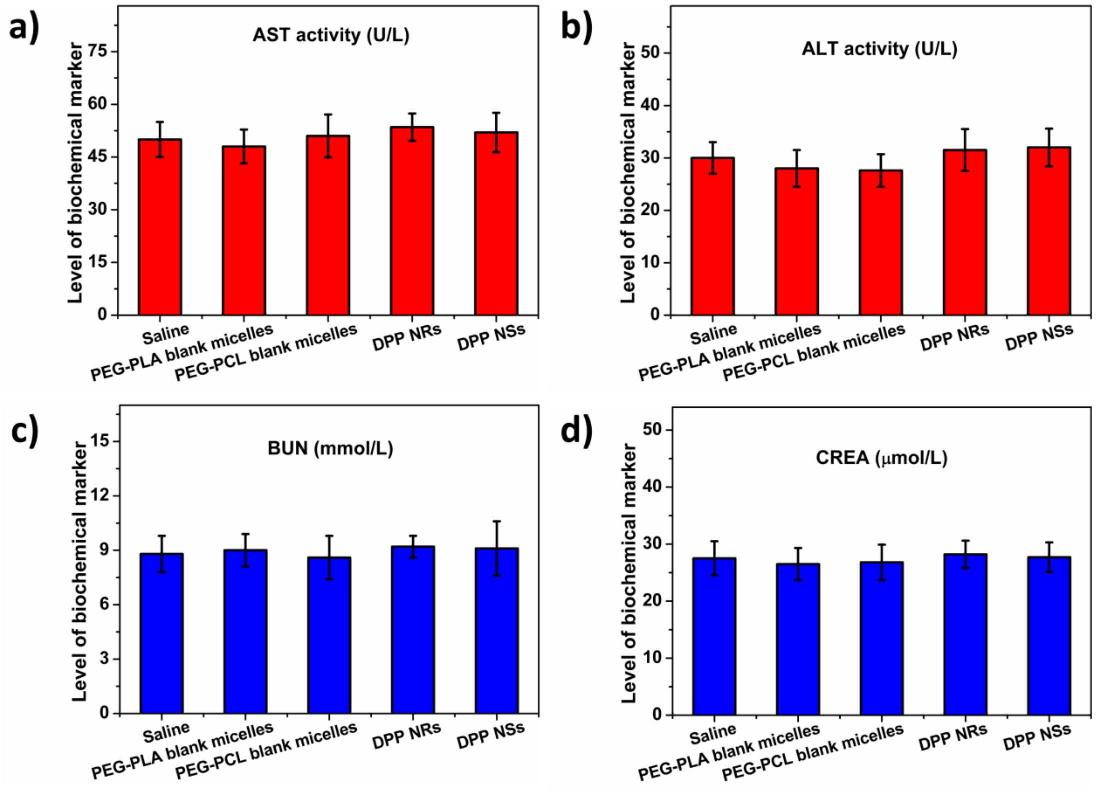

Fig. S34. The biochemical analysis of the saline, PEG-PLA blank micelles, PEG-PCL blank micelles, DPP NRs and DPP NSs groups after intravenous administraion. (a-b) The blood levels of AST and ALT from control and the treated mice of different fromulation as liver function markers. (c-d) BUN and CREA levels in the blood as the kidney function markers. Data were given as the mean ± SD (n=3).
